# Essential role of the amino-terminal region of Drosha for the Microprocessor function

**DOI:** 10.1016/j.isci.2023.107971

**Published:** 2023-09-20

**Authors:** Amit Prabhakar, Song Hu, Jin Tang, Prajakta Ghatpande, Giorgio Lagna, Xuan Jiang, Akiko Hata

**Affiliations:** 1 Cardiovascular Research Institute, University of California, San Francisco, San Francisco, CA 94143, USA; 2 Molecular Cancer Research Center, Sun Yat-Sen University School of Medicine, Guangzhou 511400, P.R.China; 3 Department of Cellular and Molecular Pharmacology, University of California, San Francisco, San Francisco, CA 94143, USA; 4 Department of Biochemistry and Biophysics, University of California, San Francisco, San Francisco, CA 94143, USA

**Keywords:** Molecular biology, Omics

## Abstract

Drosha is a core component of the Microprocessor complex that cleaves primary-microRNAs (pri-miRNAs) to generate precursor-miRNA and regulates the expression of ∼80 ribosomal protein (RP) genes. Despite the fact that mutations in the amino-terminal region of Drosha (Drosha-NTR) are associated with a vascular disorder, hereditary hemorrhagic telangiectasia, the precise function of Drosha-NTR remains unclear. By deleting exon 5 from the Drosha gene and generating a Drosha mutant lacking the NTR (ΔN), we demonstrate that ΔN is unable to process pri-miRNAs, which leads to a global miRNA depletion, except for the miR-183/96/182 cluster. We find that Argonaute 2 facilitates the processing of the pri-miR-183/96/182 in ΔN cells. Unlike full-length Drosha, ΔN is not degraded under serum starvation, resulting in unregulated RP biogenesis and protein synthesis in ΔN cells, allowing them to evade growth arrest. This study reveals the essential role of Drosha-NTR in miRNA production and nutrient-dependent translational control.

## Highlights

• Mutations in the Drosha amino-terminal region (NTR) lead to a vascular disorder
• Drosha missing the NTR (ΔN) fails to generate miRNAs except miR-183/96/182
• Ago 2 facilitates the processing of miR-183/96/182 in ΔN cells
• ΔN deregulates ribosomal protein synthesis and mediates aberrant translation

## Graphical abstract

**Figure:**
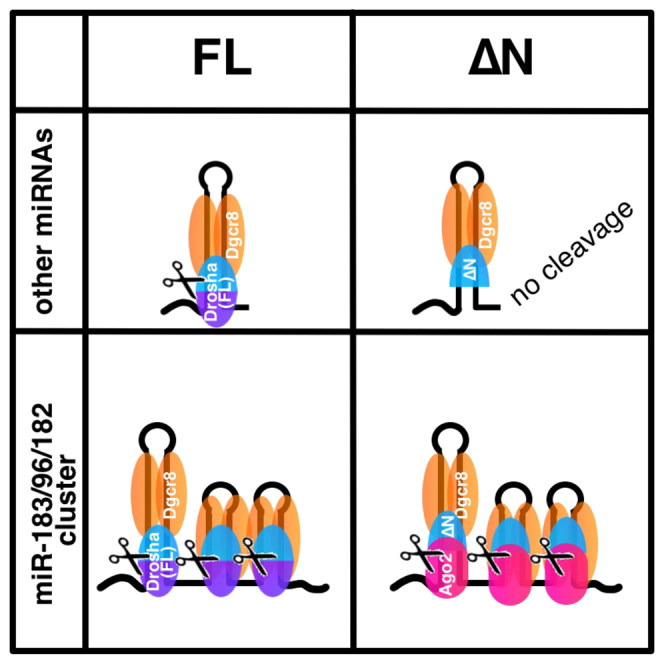

## Introduction

Drosha and DiGeorge syndrome critical region 8 (Dgcr8) are components of the Microprocessor, a complex responsible for the biogenesis of miRNAs.^1^^,^^2^ The RNase III enzyme Drosha processes long primary-miRNAs (pri-miRNAs) to generate precursor-miRNAs (pre-miRNAs) with hairpin structures in the nucleus,^1^^,^^2^ which then undergo secondary processing by Dicer in the cytoplasm to generate small RNA duplexes of ∼22-nucleotides (nt).^3^ The RNA duplexes are then loaded onto an Argonaute (Ago) protein to form an RNA-induced silencing complex (RISC), which unwinds RNA duplexes, removes the passenger strands, binds 3′UTR of mRNAs through the sequence element that is partially complementary to the miRNA sequence, and mediates destabilization and translational repression of target mRNAs.^3^ The Microprocessor-mediated processing is regulated by physiological stimuli, for example, upon activation of the TGF-β-family of growth factor signaling.^4^ All four Ago proteins (Ago1-4) incorporate miRNAs in RISC, but Ago2 is distinct because it is capable of miRNA-directed target RNA cleavage through its intrinsic RNA slicing activity.^5^ It has also been found that Ago2, rather than Dicer, processes pre-miR-451,^6^^,^^7^^,^^8^ suggesting that the slicing activity of Ago2 has broader functions beyond miRNA-mediated mRNA silencing.

In the carboxyl (C)-terminal region (CTR) of Drosha [amino acid (aa) 875-1374], two RNase III domains (aa 875-1056 and aa 1107-1233) and one double-stranded RNA-binding domain (dsRBD) (aa 1260-1344) are essential for the processing of pri-miRNAs.^1^ Conversely, the functions of the conserved proline (P)-rich region (aa 69-164) and arginine/serine (RS)-rich region (aa 217-315) in the amino (N)-terminal region (NTR) of Drosha remain to be elucidated.^1^ Given that aa 390-1365 of Drosha is sufficient to interact with Dgcr8 and process pri-miRNAs *in vitro*, the Drosha-NTR was considered dispensable for pri-miRNA processing,^9^ hence it has been incompletely studied. Several disease-associated alleles of Drosha have been identified in humans, including missense mutations in the RNase III domains of Drosha in patients with Wilms tumor^10^^,^^11^^,^^12^^,^^13^^,^^14^^,^^15^ and missense mutations in the P-rich and the RS-rich regions of Drosha (e.g., P100L and R279L) in patients with hereditary hemorrhagic telangiectasia (HHT).^16^^,^^17^ While Wilms tumor mutations in the Drosha-CTR impair the RNase III activity,^10^^,^^11^^,^^12^^,^^13^^,^^14^^,^^15^ the effect of HHT mutations in the Drosha-NTR are yet-to-be explored. We have demonstrated an important role of the Drosha-NTR in the control of ribosomal protein (RP) synthesis upon nutrients deprivation.^18^ The Microprocessor complex associates with the 5′-oligopyrimidine (5′TOP) tract of nascent RP gene (RPG) transcripts and facilitates RNA polymerase II (RNAPII) elongation via the RNA helicase Ddx5, an auxiliary component of the Microprocessor.^18^ Upon nutrient starvation, Drosha is degraded by a proteasome-dependent mechanism, which results in repression of RP synthesis, reduction of ribosomes, and inhibition of translation.^18^ Here, we expand the analysis of the Drosha-NTR by generating cell lines expressing a Drosha-NTR truncation mutant (ΔN-Drosha) from the endogenous locus. ΔN-Drosha localizes to the nucleus and interacts with Dgcr8, but it is defective in pri-miRNA processing and leads to globally diminished miRNA levels except for the miR-183/96/182 cluster. We found that the processing of pri-miR-183/96/182 (hereafter referred to as pri-miR-183) is mediated by Ago2. Finally, in response to nutrient deprivation, ΔN-Drosha is resistant to the ubiquitin-dependent degradation, and therefore, ΔN-Drosha cells do not repress RP synthesis, global translation, and cell proliferation. Our study sheds light on the essential role of the Drosha-NTR in the canonical and noncanonical functions of the Microprocessor.

## Results

### Establishing cell lines expressing Drosha without the NTR

**Figure 1:**
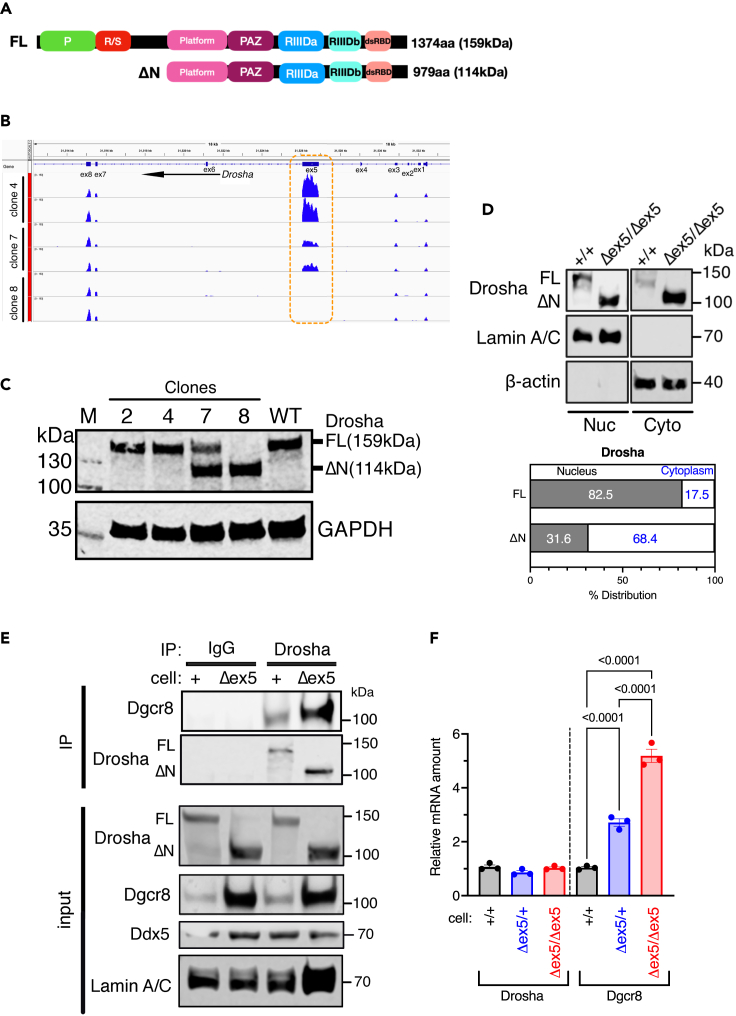
Generation of cell lines expressing Drosha truncated in the NTR (A) Schematic diagram of the domain structure of Drosha FL and ΔN-Drosha protein and the human Drosha gene structure (light blue). P: Pro-rich region, R/S: Arg/Ser-rich region, PAZ: Piwi Argonaut and Zwille domain, RIIID: RNase III domain, dsRBD: double-stranded RNA binding domain. (B) RNA-seq data of clones 4, 7, and 8 corresponding to the ex1-8 of the Drosha gene are shown. Each clone was sequenced twice. (C) Total cell lysates of clones 2, 4, 7, 8 or the original HEK293T cells (WT) were subjected to immunoblot analysis of Drosha and GAPDH (loading control). (D) Nuclear (Nuc) and cytoplasmic (Cyto) fraction of +/+ cells (FL) and Δex5/Δex5 cells (ΔN) cells were subjected to immunoblot analysis of Drosha, Lamin A/C (control for the Nuc fraction), and β-actin (control for the Cyto fraction) (top). Relative distribution (%) of FL and ΔN-Drosha in the nucleus vs. cytoplasm is shown (bottom). (E) Co-immunoprecipitation of Drosha (FL or ΔN-Drosha) and Dgcr8 in nuclear extracts of +/+ cells (+) and Δex5/Δex5 cells (Δex5). As control, non-specific IgG (control) was applied. The amount of Drosha after IP is shown. Input samples were subjected to immunoblot analyses of Drosha, Dgcr8, Ddx5, and Lamin A/C (loading control). (F) The level of the Drosha and Dgcr8 mRNA relative to GAPDH mRNA in +/+ cells (black), Δex5/+ cells (blue), and Δex5/Δex5 cells (red) were measured by qRT-PCR and plotted as mean ± SEM. n = 3 independent experiments. See also and Figures S1–S3.

The Drosha-NTR is composed of P-rich and R/S-rich regions with unclear functions (Figure 1A). To study the function of the Drosha-NTR, we generated cell lines expressing Drosha mutants lacking the NTR from the endogenous locus. In the human Drosha gene, exon 5 (ex5) encodes aa 7-284, which comprise the P-rich region (aa 69-164) and most of the R/S-rich region (aa 217-315). To generate a cell line in which ex5 of *Drosha* is deleted, we transfected two guide RNAs (gRNA1 and gRNA2) that target the intron upstream and downstream of ex5, respectively, together with the plasmid encoding the Cas9 enzyme (Figure S1). We isolated 10 stable clones, examined the status of ex5 by PCR analysis, and identified the heterozygous clone 7 (with one *Drosha* allele lacking ex5; Δex5/+) and the homozygous mutant clone 8 (with both *Drosha* alleles lacking ex5; Δex5/Δex5) (Figures S1 and S2). RNA-seq data detected no reads (in clone 8) and a smaller number of reads corresponding to ex5 in clone 7 (Figure 1B, orange box). Clones 2 and 4 retained both *Drosha* wild type alleles (+/+) (Figure S1). The translation of the Δex5 allele in clones 7 and 8 is predicted to start at methionine-396 to generate a mutant Drosha lacking the NTR (ΔN-Drosha; aa 396-1374) with a molecular weight (M.W.) of 114 kDa. Immunoblot analysis confirmed that clones 2 and 4 (+/+) express a full length (FL; aa 1-1374) Drosha (FL) with an M.W. of 159 kDa identical to the wild-type protein in the original HEK293T cells (WT) (Figure 1C) while clone 8 (Δex5/Δex5) expresses only ΔN-Drosha (Figure 1C). Clone 7 (Δex5/+) expressed both FL and ΔN-Drosha (Figure 1C), confirming its heterozygosity. To examine the subcellular localization of FL and ΔN-Drosha, the nuclear and cytoplasmic fractions of +/+ and Δex5/Δex5 cells were subjected to immunoblot analysis with anti-Drosha antibodies. FL-Drosha in +/+ cells was found predominantly in the nucleus while ΔN-Drosha in Δex5/Δex5 cells was predominantly in the cytoplasm (Figure 1D). We interpret this result as due to the loss of a nuclear localization signal (NLS) in the RS-rich region in ΔN-Drosha.^19^ Immunoprecipitation of Drosha followed by immunoblot with an anti-Dgcr8 antibody indicated that both FL and ΔN-Drosha were able to interact with Dgcr8 (Figure 1E). We noticed that ΔN-Drosha protein was 2.5-fold more abundant than FL, despite being expressed from the same locus (Figure 1E). Furthermore, Dgcr8 protein in Δex5/Δex5 cells was 8-fold more abundant than in +/+ cells (Figure 1E, input). qRT-PCR analysis showed that Drosha mRNA in +/+, Δex5/+, and Δex5/Δex5 cells was similarly expressed (Figure 1F, Drosha), indicating that the higher amount of ΔN-Drosha protein in Δex5/Δex5 cells is likely due to increased protein stability. Unlike Drosha mRNA, the amount of Dgcr8 mRNA in Δex5/Δex5 cells was 5-fold higher than in +/+ cells (Figure 1F, Dgcr8; Figure S3). Considering that Dgcr8 mRNA is cleaved by Drosha and degraded,^20^ the increased amount of Dgcr8 mRNA in Δex5/Δex5 cells suggests a defective catalytic activity of ΔN-Drosha.

### Global depletion of miRNAs in ΔN-Drosha cells

**Figure 2:**
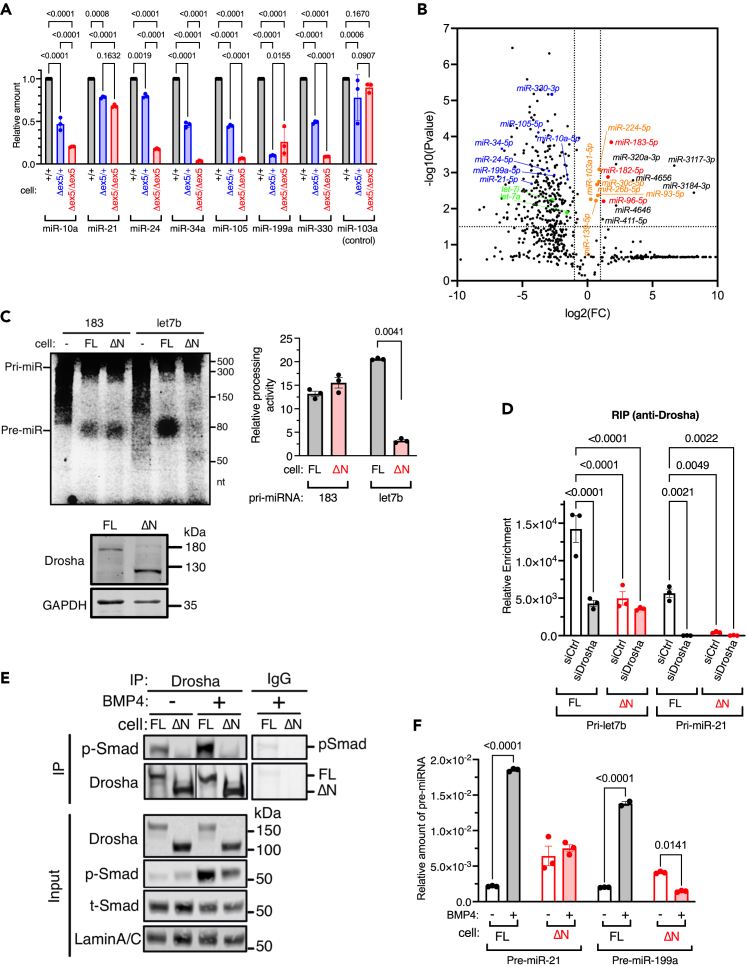
The NTR of Drosha is essential for the processing of pri-miRNAs (A) qRT-PCR analysis showing the amount of miR-10, -21, -24, -34a, -103a1, -105, -199a, and -330 relative to U6 snRNA in +/+ cells (black), Δex5/+ cells (blue), and Δex5/Δex5 cells (red). miR-103a1 is an intronic miRNA. The result was plotted as means ± SEM. n = 3 independent experiments. (B) Global analysis of miRNA expression in +/+ (clone 4) and Δex5/Δex5 (clone 8) cells is visualized in volcano plot. The fold change (FC) of miRNAs (log_2_FC) are shown in X axis and p values [-log10(P-value)] in Y axis. miRNAs analyzed in Figure 2B and let-7 family of miRNAs are shown in blue and green, respectively. Intronic miRNAs and miR-183 cluster of miRNAs (miR-182, miR-96, and miR-183) are shown in orange and red, respectively. n = 4 for clone 4 and n = 5 for clone 8. (C) The processing activity of FL- and ΔN-Drosha were examined by IVP assay by incubating pri-let7b or pri-miR-183 with whole cell lysates from FL (+/+) or ΔN (Δex5/Δex5) cells, or without cell lysates (−). Immunoblot analysis of Drosha indicates the amount of FL- and ΔN-Drosha added to the reaction (bottom). The processing products (pre-miRNAs) were separated from the substrates (pri-miRNAs) by PAGE, shown in duplicate (top left). The processing activity, after being normalized by the Drosha protein amount, is presented as mean ± SEM (top right) from 3 independent experiments. (D) Association of Drosha with pri-let7b or pri-miR-21 was assessed by RIP assay in FL cells (black) and ΔN cells (red). The amount of pri-miRNAs in the anti-Drosha antibody immunoprecipitates (Drosha IP) or non-specific IgG immunoprecipitates (IgG IP) was quantitated by qRT-PCR in triplicates. Relative enrichment of Drosha IP over IgG IP was plotted as mean ± SEM. (E) Co-immunoprecipitation of Drosha and phosphorylated-Smad1/5/8 (*p*-Smad) was examined in FL cells or ΔN cells with or without 1 nM BMP4 treatment for 2 h. As control, non-specific IgG (control) was applied. The amount of Drosha after IP is also shown. Input samples were subjected to immunoblot analyses of Drosha, *p*-Smad, total-Smad1 (t-Smad), and Lamin A/C (loading control). (F) qRT-PCR analysis of pre-miR-21 and pre-miR-199a in FL cells or ΔN cells cells with or without 1 nM BMP4 stimulation for 2 h. The result is plotted as mean ± SEM. n = 3 independent experiments.

qRT-PCR quantification of different miRNAs in +/+, Δex5/+, and Δex5/Δex5 cells showed that the levels of miR-10a, -21, -24, -34a, -105, -199a, and -330 were greatly reduced in Δex5/Δex5 cells (Figure 2A, black vs. red) and lesser degree reduced in Δex5/+ cells (Figure 2A, black vs. blue) with the exception for miR-103a, an intronic miRNA whose processing is Drosha-independent^21^(Figure 2A). When global miRNA expression was analyzed in +/+ and Δex5/Δex5 cells by small RNA-seq, out of 375 miRNAs that were differentially expressed, 357 miRNAs (95%) were lower in Δex5/Δex5 cells than +/+ cells while only 15 miRNAs (4%) were higher in Δex5/Δex5 cells (Figure 2B). Among 357 miRNAs that were reduced in Δex5/Δex5 cells, 351 miRNAs (98.3%) were > 2-fold lower in Δex5/Δex5 cells, which included those accessed in Figure 2A (Figure 2B, blue) and the let-7 family of miRNAs (Figure 2B, green), indicating a detrimental effect of the NTR deletion on the processing activity of Drosha. Only 9 out of 15 miRNAs (miR-96-5p, -182-5p, -183-5p, -320a-5p, -411-5p, -3117-3p, -3184-3p, -4646, and -4656) were > 2-fold higher in Δex5/Δex5 than +/+ cells (Figure 2B, red and black). It is of note that among 9 miRNAs higher in Δex5/Δex5 cells include 3 miRNAs in the miR-183 cluster (miR-96-5p, -182-5p, -183-5p)(Figure 2B, red). Six out of 11 miRNAs whose levels were similar between Δex5/Δex5 cells and +/+ cells were intronic-miRNAs (miR-26b-5p, -30c-5p, -93-5p, -103a1-5p, -139-5p, and -224-5p)^21^ (Figure 2B, orange), confirming that the processing of the intronic-miRNAs are independent of Drosha. An *in vitro* processing (IVP) assay was performed by incubating a fluorescence dye-labeled pri-miR-183 or pri-let7b with whole cell lysates of either +/+ cells expressing FL-Drosha (hereafter referred to as FL cells), or Δex5/Δex5 cells expressing ΔN-Drosha (hereafter referred to as ΔN cells) (Figure 2C, top). The processing of pri-let7b by ΔN-Drosha was 85% lower than that by FL-Drosha after normalizing the Drosha amount, confirming the deletion of the Drosha-NTR decreases the Microprocessor activity (Figure 2C, top right). In contrast to pri-let7b, there was no difference in the pri-miR-183 processing between ΔN-Drosha and FL-Drosha (Figure 2C, top left). This result is consistent with the small RNA-seq data, which demonstrates that no reduction in the levels of the miR-183 cluster of miRNAs in ΔN cells compared to FL cells (Figure 2B, red). The RNA-immunoprecipitation (RIP) assay showed that the association of ΔN-Drosha with the pri-let7b or pri-miR-21 hairpin was reduced by 70% and 99% compared to FL-Drosha, respectively (Figure 2D). These results not only imply that the Drosha-NTR is crucial for the stable association of Drosha with pri-miRNAs and their cleavage, but also suggest that the processing of miR-183/96/182 hairpins may be catalyzed by an RNase distinct from Drosha.

The Microprocessor activity is regulated by various proteins that associate with the Microprocessor, such as the Smads.^4^^,^^22^ Smads 1, 5, and 8, the signal transducers of the bone morphogenetic proteins (BMPs) signaling pathway, interact with Drosha upon BMP4 stimulation and promote the processing of specific miRNAs, such as miR-21 and miR-199a.^23^ IP-immunoblot analyses showed that Smad1/5/8 proteins, phosphorylated by the BMP receptor kinase upon BMP4 stimulation (*p*-Smad), associated with FL-Drosha but not with ΔN-Drosha (Figure 2E, IP), although *p*-Smads were detected in the input samples of ΔN cells stimulated with BMP4 (Figure 2E, Input). In FL cells, the levels of pre-miR-21 and pre-miR-199a increased 9-fold and 7-fold, respectively, upon BMP4 stimulation (Figure 2F, black bars). However, neither pre-miR-21 nor pre-miR-199a showed any increase in ΔN cells after BMP4 treatment (Figure 2F, red bars). These results indicate the Drosha-NTR is essential for the BMP4-dependent increase in Microprocessor activity through association with Smad proteins.

### Increased production of RPs in ΔN cells

**Figure 3:**
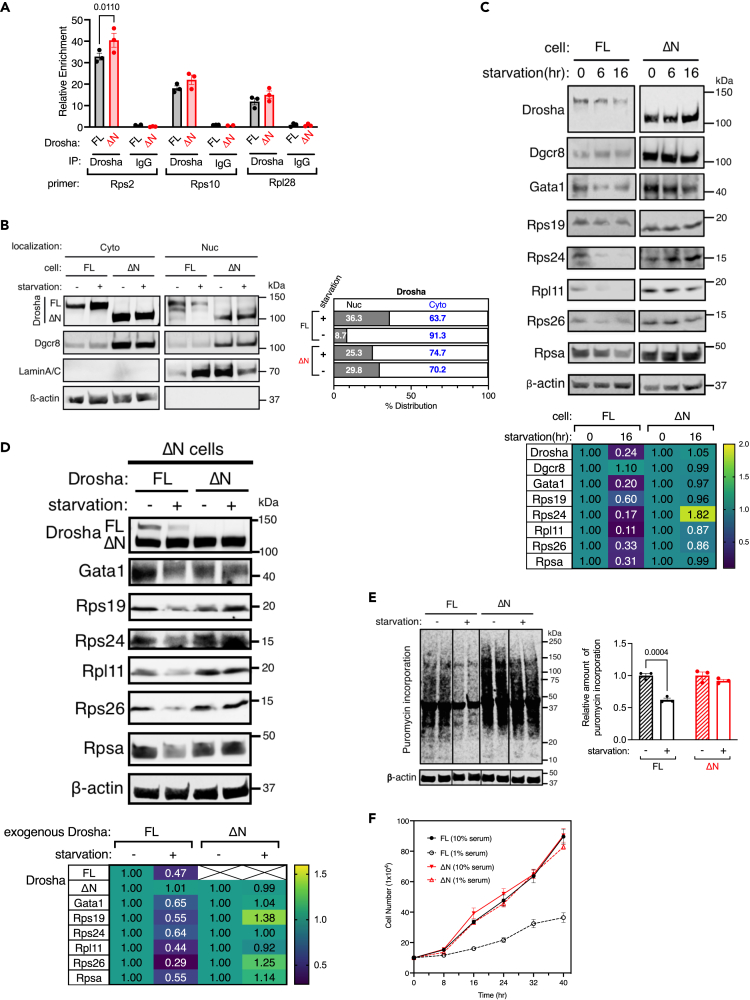
Maintenance of ribosomal protein abundance, protein synthesis, and growth in ΔN-Drosha cells under serum starvation (A) Association of Drosha with the *Rps2*, *Rps10*, and *Rpl28* transcripts. ChIP-qPCR analysis was performed by using anti-Drosha antibody (Drosha) or nonspecific IgG (IgG; negative control) in FL or ΔN-Drosha cells. The result is shown as a fold enrichment over input (Mean ± SEM). n = 3 independent experiments. (B) FL or ΔN-Drosha cells were treated with or without serum starvation (1%) for 16 h, followed by the preparation of cytoplasmic (Cyto) and nuclear (Nuc) lysates and subjected to immunoblot for Drosha, Dgcr8, Lamin A/C (loading control for the Nuc fraction) and β-actin (loading control for the cytoplasmic fraction) (left). The immunoblot result is quantitated and % distribution of FL or ΔN-Drosha protein is shown (right). (C) FL or ΔN cells were serum starved (1% serum) for 0, 6, or 16 h, followed by immunoblot analysis for Drosha, Dgcr8, Gata1, RPs (Rps19, Rps24, Rpl11, Rps26, Rpsa), and β-actin (loading control) (top). The relative protein amounts at 0 and 16 h starvation normalized to β-actin are shown in a heatmap (bottom). (D) ΔN cells, in which either FL-Drosha (FL) or ΔN-Drosha (ΔN) was exogenously expressed, with or without serum starvation (1% serum) for 16 h were subjected to immunoblot analysis of Drosha, Gata1, RPs (Rps19, Rps24, Rpl11, Rps26, and Rpsa) (top). The protein amounts normalized to β-actin are shown in a heatmap (bottom). (E) FL or ΔN-Drosha cells were treated with or without serum starvation (1% serum) for 16 h, followed by puromycin treatment for 10 min and immunoblot analysis with anti-puromycin antibody and anti-β-actin antibody (loading control) (left). Each condition is shown in duplicate (left). The relative abundance of puromycin-incorporated proteins normalized by β-actin is shown as mean ± SEM (right). n = 3 per group. Unpaired two-tail t-test was used for the statistical analysis. (F) FL or ΔN-Drosha cells were cultured in growth media (10% serum) or starvation media (1% serum) and the cell number was counted at 8, 16, 24, 32, and 40 h after the media change. The result is plotted as mean ± SEM. n = 5 independent samples. See also Figure S4.

The Microprocessor potentiates the transcription of RPGs by binding to the 5′TOP sequence shared by all ∼80 RPGs.^18^ A ChIP assay indicated that both FL- and ΔN-Drosha associate with the *Rps2*, *Rps10*, and *Rpl28* gene loci at similar levels (Figure 3A), indicating that the Drosha-NTR is dispensable for interaction with RPGs. We showed that, upon serum starvation, Drosha translocates from the nucleus to the cytoplasm and is degraded through the association of an E3 ubiqutin ligase Nedd4 at Drosha-NTR, resulting in the repression of RPG transcription.^18^ Consistently, the nuclear localization of FL-Drosha decreased from 36.3% to 8.7%, however, ΔN-Drosha remained in the nucleus upon serum starvation (Figure 3B). Furthermore, 16 h after serum starvation, the amount of FL-Drosha decreased to 24% (Figure 3C). The levels of both RPs (Figure 3C) and RP mRNAs (Figure S4A) in FL cells also decreased after starvation as previously reported.^18^ However, the amount of ΔN-Drosha remained the same after starvation (Figure 3C). Furthermore, the levels of RPs (Figure 3C) and RP mRNAs (Figure S4A) remained high in ΔN cells after starvation. The amount of Gata1 protein, which is sensitive to changes in ribosome abundance,^18^ was reduced by 80% in FL cells after serum starvation (Figure 3C). In ΔN cells, however, Gata1 protein decreased only by 3% (Figure 3C). This is consistent with the absence of the reduction of RPs under starvation (Figure 3C). When FL-Drosha was exogenously expressed in ΔN cells, a reduction of FL-Drosha, RPs (Figure 3D), and RP mRNAs (Figure S4B) was observed following serum starvation. This indicates that exogenous FL-Drosha is capable of rescuing the control of RP biogenesis in ΔN cells. In contrast, when ΔN-Drosha was introduced in ΔN cells, the levels of RPs (Figure 3D), RP mRNAs (Figure S4B) and ΔN-Drosha remained unchanged under starvation. These results underscore the essential role of Drosha-NTR in controlling RP biosynthesis in response to changes in nutrient availability. An *in vitro* puromycin incorporation assay^24^ showed that the amount of puromycin-incorporated nascent proteins was reduced to 38% in FL cells after serum starvation for 16 h (Figure 3E), indicating a reduced translation. In contrast, there was no decrease in translation upon starvation in ΔN cells (Figure 3E), which aligns with the continued RP biogenesis in ΔN cells during serum starvation (Figure 3C). When the proliferation of FL and ΔN cells was compared under normal growth conditions (10% serum) and starvation conditions (1% serum), the doubling time (*Td*) of FL cells increased from 20 h (10% serum) to 56 h (1% serum) (Figure 3F, black lines), indicating slower proliferation under serum starvation conditions. In contrast, ΔN cells proliferated at a consistent rate in both 10% serum (*Td* = 21 h) and 1% serum (*Td* = 21 h) conditions (Figure 3F, red lines). These results demonstrate that while the Drosha-NTR is dispensable for the association with RPGs, it is essential for starvation-induced degradation of Drosha. Consequently, ΔN cells cannot modulate ribosome abundance and cell proliferation rate to adapt to the changes in the environment.

### Ago2-dependent processing of the miR-183 cluster

**Figure 4:**
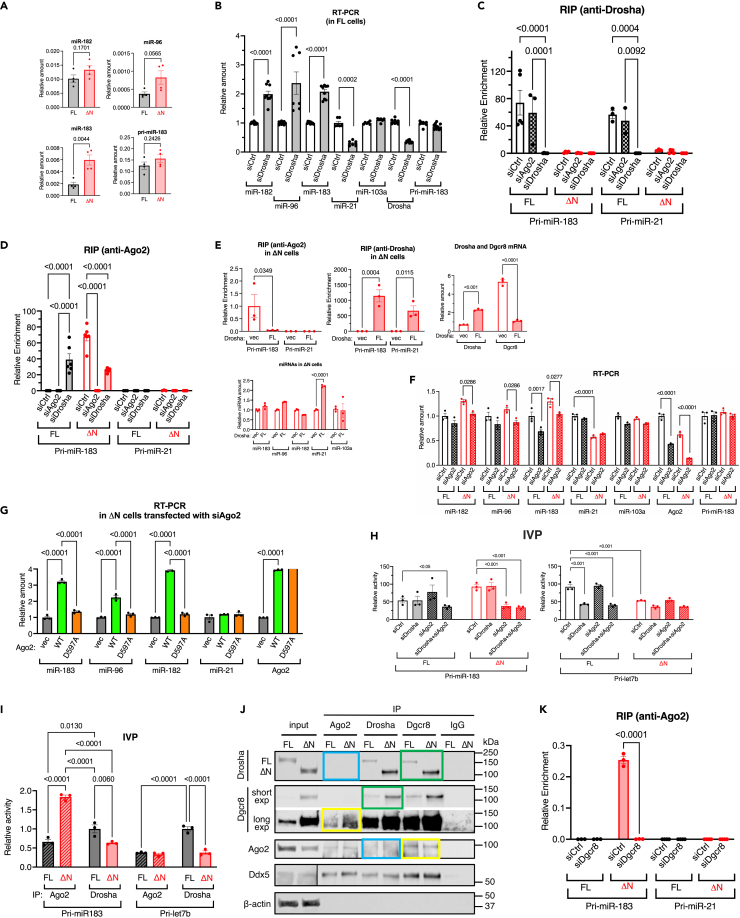
Ago2-dependent processing of miR-183 cluster (A) qRT-PCR analysis of miR-182, -96, and -183 (normalized to U6 snRNA), and pri-miR-183 (normalized to GAPDH) in FL and ΔN-Drosha cells. The result is plotted as mean ± SEM. n = 4 independent experiments. (B) FL cells transfected with siRNA against non-specific control (siCtrl) or Drosha (siDrosha) were subjected to qRT-PCR analysis of miR-182, -96, -183, control miRNAs (miR-21, and -103a) (normalized to U6 snRNA), Drosha mRNA, and pri-miR-183 (normalized to GAPDH) was performed in triplicates. The result is plotted as mean ± SEM. n = 2–3 independent experiments. (C) RIP assay to access the interaction between Drosha (FL or ΔN) and pri-miR-183 or pri-miR-21 in FL and ΔN-Drosha cells transfected with siCtrl, siAgo2, or siDrosha. The amount of pri-miR-183 or pri-miR21 in the immunoprecipitates (IP) of anti-Drosha antibody or non-specific IgG (control) was quantitated by qRT-PCR in triplicates. The relative enrichment of pri-miR-183 or pri-miR21 (Drosha IP/IgG IP) was plotted as mean ± SEM. n = 2–3 independent experiments. (D) Association of Ago2 with pri-miR-183 or pri-miR-21 was assessed by RIP assay in FL and ΔN-Drosha cells transfected with siCtrl, siAgo2, or siDrosha. The amount of pri-miR-183 or pri-miR-21 in the IP of anti-Ago2 antibody or non-specific IgG (control) was quantitated by qRT-PCR in triplicates. The relative enrichment of pri-miR-183 or pri-miR-21 (Drosha IP/IgG IP) is plotted as mean ± SEM. n = 2–3 independent experiments. (E) ΔN-Drosha cells were transfected with an empty vector (vec) or FL-Drosha expression plasmid (FL), followed by RIP assay with IP with anti-Ago2 antibody (top left) or anti-Drosha antibody (top middle). As negative control for RIP, non-specific IgG was used. The amount of pri-miR-183 or pri-miR-21 in the IP was quantitated by qRT-PCR and the relative enrichment of pri-miR-183 or pri-miR-21 (Ago2 IP/IgG IP or Drosha IP/IgG IP) was plotted as mean ± SEM. qRT-PCR of Drosha and Dgcr8 mRNA amount relative to GAPDH is shown (top right). n = 3. qRT-PCR analysis of miR-183, -96, -182, control miRNAs (miR-21, and -103a) (normalized to U6 snRNA) was performed in triplicates (bottom). n = 3. (F) qRT-PCR of miR-182, -96, 183, -21, and -103a, Ago2, and pri-miR-183/96/182 in FL or ΔN-Drosha cells transfected with siCtrl or siAgo2. The amount of miRNAs and Drosha mRNA normalized to U6 snRNA and GAPDH, respectively, and plotted as mean ± SEM. n = 3 independent experiments. (G) ΔN cells depleted in Ago2 with siAgo2 were transfected with an empty vector (vec) or an expression plasmid carrying wild type Ago2 (WT) or noncatalytic Ago2 mutant (D597A) and the amount of miR-183, -96, -182, and -21 (control) normalized to U6 snRNA and Ago2 mRNA normalized to GAPDH were examined by qRT-PCR. The result is presented as mean ± SEM. n = 3. (H) The IVP assay of pri-let7b and pri-miR-183 incubated with cell lysates of FL or ΔN cells transfected with siCtrl, siDrosha, siAgo2, or siDrosha+siAgo2. The relative processing activity was quantitated, normalized by Drosha amount, and shown as mean ± SEM (bottom). n = 3. (I) The IVP assay of pri-let7b and pri-miR-183 incubated with immnopurified Ago2 or Drosha from FL or ΔN cells. The relative processing activity is shown as mean ± SEM (bottom). n = 3. (J) Ago2, Drosha, Dgcr8 were immunoprecipitated in FL and ΔN-Drosha cells, followed by immunoblot with anti-Drosha, anti-Dgcr8, anti-Ago2, or anti-Ddx5 antibody. Drosha-Dgcr8 (green boxes) and Dgcr8-Ago2 (yellow boxes) interaction was detected while Drosha-Ago2 interaction was not detected (blue boxes). Non-specific IgG was used as control IP. Anti-β-actin antibody for input samples was used as loading control. (K) FL and ΔN-Drosha cells were transfected with siCtrl or siDgcr8, followed by RIP assay with IP with anti-Ago2 antibody or non-specific IgG (control). The amount of pri-miR-183 or pri-miR-21 in the IP was quantitated by qRT-PCR and the relative enrichment of pri-miR-183 or pri-miR-21 (Ago2 IP/IgG IP) was plotted as mean ± SEM. n = 3. See also Figures S5–S11 and S13.

Among a small number of miRNAs expressed in ΔN cells at a level equivalent or slightly higher than FL cells were miR-183, -96, and -182 (Figure 2B, left; Figures S5A and S5B). These miRNAs belong to the miR-183 cluster and are transcribed as a single long polycistronic transcript (pri-miR-183) with three hairpin structures corresponding to mature miR-183, -96, and -182.^25^ qRT-PCR confirmed the small RNA-seq data that the levels of these miRNAs were slightly higher in ΔN cells compared to FL cells (Figure 4A). Consistently, we observed reduced mRNA levels of targets of miR-183/96/182 in ΔN cells compared to FL cells (Figure S5C).^25^ The levels of pri-miR-183 transcripts were similar in FL and ΔN cells (Figure 4A), indicating no difference in the miR-183 cluster gene transcription between FL and ΔN cells. When Drosha was depleted by siRNA (siDrosha) in FL cells, the levels of miR-183 cluster miRNAs were slightly elevated (Figure 4B) while the levels of control miRNAs (miR-21, -24, -105, and -330) were diminished (Figure 4B; Figure S6). The small RNA-seq data revealed a large fraction of miR-183 and miR-96 sequence variants (isomiRs) (Table 1), which contain 1 or 2 additional nucleotides at the 5′-end with respect to the reference sequence (Table 1), in ΔN cells, suggesting that the cleavage of these pri-miRNAs in ΔN cells occurred 1- or 2-nt upstream of the conventional Drosha cleavage site. Unlike miR-183 and miR-96, we did not find 5′-end sequence variants of miR-21, miR-10a, miR-34a, and miR-105 in FL or ΔN cells (Table 1). Together with the result of IVP assay (Figure 2C), these results support the hypothesis that an enzyme other than Drosha processes hairpins in pri-miR-183 when Drosha is depleted or unable to bind pri-miRNA like ΔN-Drosha.

**Table 1:** IsomiRs of miR-183 and miR-96 are found in ΔN-Drosha cells

miRNA	Sequence	Length (nt)	FL (RPM)	ΔN-Drosha (RPM)	Ratio (ΔN-Drosha/FL)
	**UG**UAUGGCACUGGUAGAAUUCACU	24	4.653	50.75	10.9
**miR-183-5p**	**G**UAUGGCACUGGUAGAAUUCACU	23	1.338	9.896	7.4
	UAUGGCACUGGUAGAAUUCACU	22	1566	2122	1.4
	**-**AUGGCACUGGUAGAAUUCACU	21	353.5	543.1	1.5
	**UU**UUGGCAAUGGUAGAACUCACACU	25	0	0	–
**miR-182-5p**	UUUGGCAAUGGUAGAACUCACACU	24	1486	1468	1.0
	**-**UUGGCAAUGGUAGAACUCACACU	23	0	0	–
	**U**UUUGGCACUAGCACAUUUUUGCU	24	1.218	6.41	5.3
**miR-96-5p**	UUUGGCACUAGCACAUUUUUGCU	23	612.9	619	1.0
	**-**UUGGCACUAGCACAUUUUUGCU	22	0	0	–
	**G**UAGCUUAUCAGACUGAUGUUGA	23	0	0	–
miR-21-5p	UAGCUUAUCAGACUGAUGUUGA	22	2641.9	223.6	0.08
	**-**AGCUUAUCAGACUGAUGUUGA	21	0	0	–
	**A**UACCCUGUAGAUCCGAAUUUGUG	24	0	0	–
miR-10a-5p	UACCCUGUAGAUCCGAAUUUGUG	23	6310.2	4760.9	0.75
	**-**ACCCUGUAGAUCCGAAUUUGUG	22	9750.7	2808.1	0.29
	**U**UGGCAGUGUCUUAGCUGGUUGU	23	0	0	–
miR-34a-5p	UGGCAGUGUCUUAGCUGGUUGU	22	10359.8	6107.3	0.59
	**-**GGCAGUGUCUUAGCUGGUUGU	21	204.5	0	–
	**G**UCAAAUGCUCAGACUCCUGUGGU	24	0	0	–
miR-105-5p	UCAAAUGCUCAGACUCCUGUGGU	23	717.2	36.5	0.051
	**-**CAAAUGCUCAGACUCCUGUGGU	22	99.6	1.3	0.013

The average number reads (reads per million; RPM) of the miR-183 cluster (miR-183-5p, -182-5p, -96-5p) and control miRNAs (miR-21-5p, -10a-5p, -34a-5p, and -105-5p) in FL or ΔN-Drosha cells are shown. Reference sequences of miR-183-5p, -182-5p, -96-5p are based on the miRBase. Extra or missing nucleotides at the 5′-end in respect of the reference sequence are shown in bold. The ratio (ΔN-Drosha/FL) of the isomiR-183 that is 1-nt shorter than the reference sequence is close to the ratio of total number of miR-183-5p reads. The ratio of isomiR-183-5p (10.9 and 7.4) and isomiR-95-5p (5.3) are higher than the ratio of total number of miR-183-5p (1.4) and miR-96-5p (1.0), respectively. No isomiRs of control miRNAs was found.

Previously, high-throughput sequencing following cross-linking immunoprecipitation (HITS-CLIP) analysis detected an association of Ago2 with miR-183/96/182.^26^ Ago2 is uniquely capable of directly cleaving RNA targets^5^^,^^27^^,^^28^ and localizes in both the cytoplasm and the nucleus.^29^ We found that ∼40% of Ago2 is localized in the nucleus in both FL and ΔN cells (Figure S7). Therefore, we hypothesized that nuclear Ago2 might be processing miR-183/96/182 hairpins under the circumstance that Drosha amount or activity is compromised. As expected, the RIP assay showed an association of FL-Drosha with both pri-miR-183 and pri-miR-21 (control) in FL cells (Figure 4C, black, siCtrl). This interaction was diminished upon Drosha depletion (Figure 4C, black, siDrosha), however, Ago2 depletion did not affect the interaction in FL cells (Figure 4C, black, siAgo2). Unlike in FL cells, no association of ΔN-Drosha with pri-miR-183 or pri-miR-21 was detected in ΔN cells (Figure 4C, red, siCtrl), despite the comparable amounts of pri-miR-183 or pri-miR-21 between ΔN and FL cells (Figure S8A) and 7-fold higher amount of ΔN-Drosha than FL-Drosha was immunoprecipitated in the RIP samples (Figure S9). The RIP assay confirmed the association of Ago2 with pri-miR-183 in ΔN cells (Figure 4D, red, siCtrl). When Ago2 was depleted by siRNA (siAgo2), the RIP signal in ΔN cells (siCtrl) diminished (Figure 4D, red, siAgo2), confirming the specific detection of the Ago2-pri-miR-183 interaction by RIP. Ago2 did not associate with pri-miR-21 in either ΔN-Drosha or FL cells (Figure 4D, red, siCtrl), underscoring the specificity of the Ago2-pri-miR-183 interaction. Although similar amounts of Ago2 protein were immunoprecipitated in ΔN and FL cells (Figure S8B), the Ago2-pri-miR-183 interaction was not detected in FL cells presumably because FL-Drosha prevented the association of Ago2 with pri-miR-183 (Figure 4D, black, siCtrl). Only when FL-Drosha was depleted by siDrosha, the Ago2-pri-miR183 interaction became detectable in FL cells (Figure 4D, black, siDrosha). When exogenous FL-Drosha was introduced at 3.3-fold higher amount than the amount of ΔN-Drosha into ΔN cells (Figure 4E, top right), the Ago2-pri-miR-183 interaction was diminished (Figure 4E, top left) and, instead, the association of FL-Drosha with pri-miR-183 was detected (Figure 4E, top middle). It is also noted that the levels of Dgcr8 mRNA was reduced by the introduction of FL-Drosha in ΔN cells (Figure 4E, top right). These results indicate that FL-Drosha and Ago2 compete for pri-miR-183 binding. There was no significant change in the levels of miR-183, -96, or -182 by exogenous FL-Drosha in ΔN cells (Figure 4E, bottom), suggesting that both FL-Drosha and Ago2 are capable of processing pri-miR-183 with similar efficiency. We also detected the association of Drosha with pri-miR-21 (Figure 4E, top middle) and the amount of miR-21 was elevated 2.2-fold (Figure 4E, bottom) as expected when FL-Drosha was exogenously expressed in ΔN cells. These results indicate that exogenous FL-Drosha is capable of associating with pri-miR-21 and promoting the biogenesis of miR-21 in ΔN cells, providing evidence that ΔN cells do not lack any essential components of the Microprocessor or miRNA biogenesis pathway. Unlike Ago2, Dgcr8 interacted with both pri-miR-183 and pri-miR-21 in FL-Drosha and ΔN cells at similar levels, which were not affected by the depletion of Ago2 or Drosha (FL or ΔN) (Figures S10A and S10B), indicating the association of Dgcr8 with pri-miRNAs is independent of Drosha or Ago2. Depletion of Ago2 by siAgo2 did not alter the amount of miR-183 cluster miRNAs in FL cells (Figure 4F, black), but significantly diminished it in ΔN cells (Figure 4F, red). When wild type (WT) Ago2 was introduced to ΔN cells, in which endogenous Ago2 had been reduced by siRNA to ∼40%, the levels of miR-183, -96, and -182 increased ∼2- to 3-fold compared to cells transfected with a control vector (vec) (Figure 4G). In contrast, when the endonuclease-inactive Ago2 mutant (D597A)^27^ was introduced to ΔN cells in an amount similar to Ago2 (WT), the levels of these miRNAs remained similar to those in control cells (Figure 4G). The amount of miR-21 (control) was affected by neither Ago2 (WT) nor Ago2 (D597) (Figure 4G). These results demonstrate that the processing of the miR-183 cluster, but not other miRNAs, is dependent on the catalytic activity of Ago2. The IVP assay confirmed that the depletion of Drosha or Ago2 had no effect on the processing of pri-miR-183 in FL cells (Figure 4H; Figure S11). However, in ΔN cells, the levels of pri-miR-183 processing decreased by 50% and 82% when Ago2 and Drosha+Ago2 were depleted, respectively (Figure 4H; Figure S11). In contrast, pri-let7b (control) processing was reduced by 65% and 64% when Drosha and Drosha+Ago2 were depleted in FL cells, respectively (Figure 4H; Figure S11). When Ago2 was immunopurified from ΔN cells and added to the IVP reaction, pri-miR-183 processing was ∼2-fold more efficient compared to that by FL-Drosha (Figure 4I). In contrast, the cleavage of pri-let7b (control) by either Ago2 or ΔN-Drosha remained lower than that of FL-Drosha (Figure 4I). These results demonstrate Ago2-dependent processing of pri-miR-183 in ΔN cells.

IP-immunoblot analysis detected the Drosha (FL and ΔN)-Dgcr8 association (Figure 4J, green boxes) and the Dgcr8-Ago2 association (Figure 4J, yellow boxes), but not the Drosha-Ago2 association (Figure 4J, blue boxes). These results suggest that the association of Ago2 with pri-miR-183 is facilitated by the physical interaction between Ago2 and Dgcr8. Furthermore, RIP assay showed that the Ago2-pri-miR-183 interaction was diminished (Figure 4K, siDgcr8) when Dgcr8 was knocked down in ΔN cells (Figure S10C). These data uncover a noncanonical processing of pri-miR-183 by Ago2 and Dgcr8 when Drosha is absent or missing the NTR and unable to associate with pri-miRNAs.

### The miR-183 hairpin-dependent processing of the miR-96 hairpin

**Figure 5:**
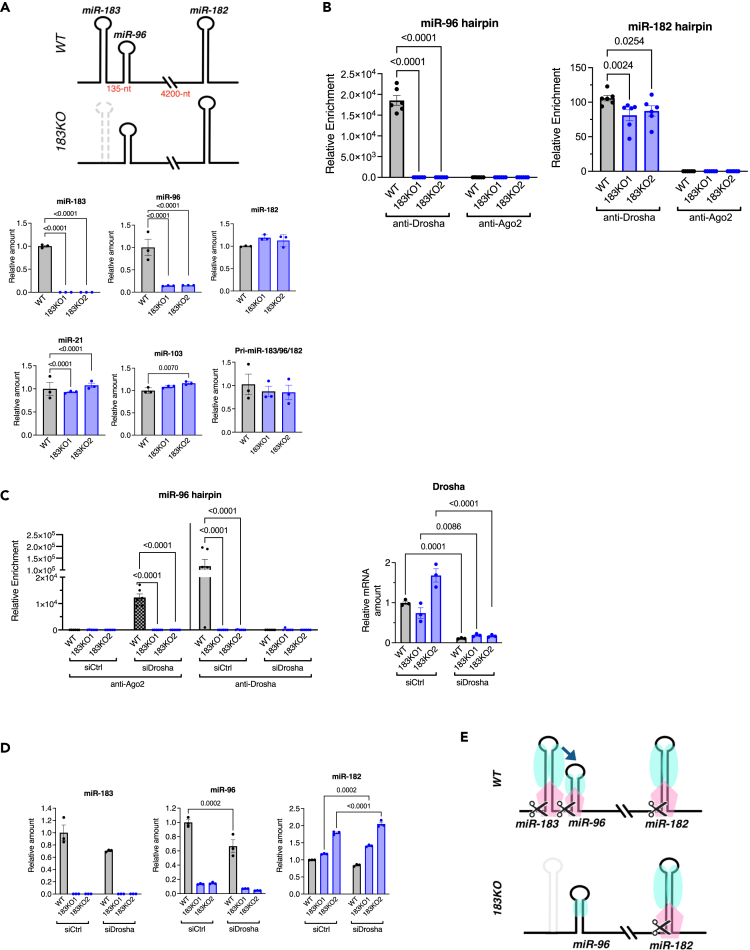
miR-183 hairpin is required for assisting the processing of miR-96 hairpin (A) The amount of miR-183, -96, -182, control miRNAs (miR-21, and -103a) (normalized to U6 snRNA) and pri-miR-183/96/182 (normalized to GAPDH) in wild type HEK293T (WT) cells and two independent miR-183 knock out clones (183KO1 and 183KO2) was quantitated by qRT-PCR in triplicates. (B) Association of Drosha or Ago2 with the miR-96 or the miR-183 hairpin was assessed by RIP assay in WT and 183KO cells. The amount of the miR-96 hairpin or the miR-183 hairpin in the IP of anti-Drosha, anti-Ago2 antibody or nonspecific IgG was quantitated by qRT-PCR in triplicates. The relative enrichment of the miR-96 hairpin or the miR-183 hairpin (Drosha IP/IgG IP or Ago2 IP/IgG IP) is plotted as mean ± SEM. n = 3. (C) Association of Drosha or Ago2 with the miR-96 hairpin or the miR-183 hairpin was assessed by RIP assay in WT and 183KO cells transfected with siCtrl or siDrosha in triplicates. The relative enrichment of the miR-96 hairpin or the miR-183 hairpin (Ago2 IP/IgG IP or Drosha IP/IgG IP) is plotted as mean ± SEM. n = 3. (D) The amount of miR-183, -96, and -182 normalized to U6 snRNA in wild type HEK293T (WT) and 183KO cells transfected with siCtrl or siDrosha was quantitated by qRT-PCR in triplicates. The graph was plotted as mean ± SEM. (E) Schematic description of the miR-183 hairpin-assisted recruitment of the Microprocessor to the miR-96 hairpin in WT cells. In the absence of the miR-183 hairpin (183KO cells), the Microprocessor (blue and pink circles) is not recruited to the miR-96 hairpin and therefore, the miR-96 hairpin cannot be processed. The miR-182 hairpin processing is independent of the miR-183 hairpin and is performed equally in WT and 183KO cells. Blue circle: Dgcr8. Pink circle: Drosha or Ago2. See also Figure S12.

According to the miRbase database and the Mfold RNA folding software, the predicted length of the stem structure in the miR-96 hairpin is 27-bp.^25^ This length is shorter than the optimal stem length of hairpins that are cleaved by Drosha (∼35 bp).^30^^,^^31^ Thus, we hypothesized that the processing of suboptimal miR-96 hairpin might be assisted by other hairpins in the same cluster. To test the hypothesis in the endogenous context, we generated two HEK293T mutants (183KO1 and 183KO2), in which biallelic deletion of the miR-183 hairpin was introduced by CRISPR-Cas9 gene editing (Figure S12), and the amounts of miR-183, -96 and -183 were examined by qRT-PCR. miR-183 was not detected in 183KO1 or 183KO2 cells, validating the deletion of the miR-183 hairpin in 183KO clones (Figure 5A). Although the level of miR-182 in 183KO clones was similar to that in WT cells, the level of miR-96 in 183KO clones decreased to 15% of that in WT cells, even though the amount of pri-miR-183 in 183KO and WT cells was comparable (Figure 5A). These results suggest that the processing of the miR-96 hairpin is dependent on the presence of the miR-183 hairpin, which is located 135-nt apart. The association of Drosha with the miR-96 hairpin was undetectable in 183KO cells by RIP assay (Figure 5B, left). This is consistent with the depletion of miR-96 in 183KO cells (Figure 5A). Unlike the miR-96 hairpin, the miR-182 hairpin associated with Drosha in both WT and 183KO cells (Figure 5B, right), indicating that the processing of the miR-182 hairpin, which is located > 4200-nt apart from the miR-183 and miR-96 hairpins, is independent of the miR-183 hairpin. When Drosha was depleted by siDrosha, Ago2 was recruited to the miR-96 hairpin in WT cells, but not in 183KO cells (Figure 5C). In WT cells, the depletion of Drosha did not affect miR-96 levels since its processing was maintained by Ago2 (Figure 5D). However, in 183KO cells, miR-96 levels remained as low as those observed in WT cells (Figure 5D), suggesting that Ago2 requires the presence of the miR-183 hairpin to bind to and process the miR-96 hairpin similar to Drosha. These results demonstrate that the processing of the miR-96 hairpin is assisted by the miR-183 hairpin (Figure 5E), a mechanism similar to that observed in the miR-144/451 cluster.^32^

## Discussion

In this work, we demonstrated that ΔN-Drosha fails to associate with pri-miRNAs, and thus Drosha-NTR is essential for the Microprocessor activity. A previous *in vitro* study on an N-terminus truncated Drosha (ΔN-390; aa 390-1374), also lacking the P-rich region and the R/S-rich region as the ΔN-Drosha used in this study, showed that processing of pri-let7a1 by ΔN-390 was comparable to that of Drosha (WT),^9^ leading to the conclusion that the Drosha-NTR (aa 1-389) was dispensable for the catalytic activity. Based on the immunoblot presented, though, it is possible that a higher amount of ΔN-390 than the WT protein might have been used in the IVP assay, decreasing the difference in activity between the two Drosha forms.^9^ The structure of the NTR-truncated Drosha (aa 353-1372), a partial Dgcr8 (aa 223-751), and a pri-miRNA-16-2 by cryo-electron microscopy demonstrate that the basal tip of the central domain (CED; aa 353-960) of Drosha wraps around the dsRNA-single stranded RNA (ssRNA) junction of the pri-miRNA.^30^^,^^31^ Our result that ΔN-Drosha, which lacks 43 aa of the N-terminus of the CED, is defective in the association with pri-miRNAs, underscores the significance of the CED for the interaction with pri-miRNAs.

Pri-miRNA hairpin structures contain several key structural features that facilitate processing by the Microprocessor.^33^^,^^34^ Our results show that miR-96, which has a suboptimal short stem structure, requires the miR-183 hairpin to facilitate cleavage by Drosha or Ago2. This is similar to the miR-144/451cluster, where the presence of the miR-144 hairpin with an optimal structure aids in the recruitment of the Microprocessor and the cleavage of a suboptimal miR-451 hairpin; the mechanism is called “cluster assistance.”^32^ Given that the miR-144 hairpin and miR-451 hairpin are only 100-nt apart similar to the miR-183 and miR-96 hairpins that is 135-nt apart, the proximity of the two hairpins in the cluster appears to be critical, however, the exact mechanism of “cluster assisted” processing remains to be elucidated. miR-182, which has an optimal hairpin structure but is located > 4200-nt apart from the miR-183 and miR-96 hairpins, is bound and cleaved by Drosha regardless of the presence of the miR-183 hairpin and is unable to facilitate the cleavage of the miR-96 hairpin. We found that the miR-3136 hairpin, which has a long stem of 40-nt like the miR-183 and miR-182 hairpins (Figure S13A), does not associate with Ago2 in ΔN cells (Figures S13B and S13C), indicating that structural characteristics of the miR-183/96/182 cluster hairpins, other than the stem length, play an important role in the recruitment of Ago2.

The nuclear localization of Drosha is mediated by a predicted nuclear localization signal (NLS) in the R/S-rich region.^19^ An alternatively spliced form of Drosha, missing exon 6 which encodes NLS, localizes both in the nucleus and the cytoplasm.^35^^,^^36^ Furthermore, the phosphorylation of the serine (Ser)-300 or Ser-302 residue by glycogen synthase kinase 3β (GSK3β) is required for the nuclear retention of Drosha.^37^^,^^38^ We previously showed that p38 MAPK-dependent phosphorylation of Drosha at Ser-355 contributes to the nuclear-to-cytoplasmic shuttling of Drosha upon nutrient starvation.^39^ Because ΔN-Drosha is missing the NLS, Ser-300, Ser-302, and Ser-355, we predicted that ΔN-Drosha would localize exclusively in the cytoplasm; however, the results indicate that ∼70% of ΔN-Drosha is localized in the nucleus, indicating the presence of additional NLS or nuclear retention signals in aa 396-1374 of Drosha. Despite ΔN-Drosha being expressed from the endogenous Drosha loci and the mRNA amount being similar between ΔN-Drosha and FL, we noted the higher protein quantity of ΔN-Drosha than FL-Drosha. We previously reported that Drosha is degraded upon ubiquitination by Nedd4,^39^ which requires the PPGY motif at aa 169-172 located in the NTR of Drosha associating with the WW domains of Nedd4.^39^ Additionally, it has been reported that the stability of Drosha protein can be modulated by the acetylation of lysine (Lys)-48 in Drosha-NTR by p300, CBP, and GCN5, which competes with ubiquitination of the same Lys residues.^40^ Because ΔN-Drosha lacks both the PPGY motif and Lys-48, we speculate that ΔN-Drosha resists ubiquitin-proteasome-dependent degradation, resulting in a higher protein amount than FL-Drosha.

HHT is an autosomal dominant vascular disorder caused by the loss-of-expression or loss-of-function mutations in the mediators of the BMP signaling pathway, such as *Acvrl1*, *Endoglin*, and *Smad4*.^41^ Nonsynonymous mutations in the Drosha-NTR, such as P32L, P100L, K226E, and R279L, have been identified in individuals with HHT without other mutations associated with HHT.^16^ HHT patients with *Drosha* mutations exhibit a range of vascular defects stemming from abnormal vascular endothelial cell structure and functions, including epistaxis, telangiectasias, and arteriovenous malformations (AVMs).^16^ Similar to the ΔN-Drosha, Drosha mutants P100L and R279L fail to associate with Smad proteins and is unable to mediate BMP4-Smad1/5/8 dependent induction of miR-21 and miR-199a.^16^ Because ΔN-Drosha is unable to control RP biogenesis, protein synthesis, and cell proliferation upon nutrient deprivation, we speculate that the vascular phenotypes in *Drosha* mutation carrier patients result from the inability of vascular endothelial cells to generate miRNAs upon BMP stimulation and the inability to adapt to changes in the extracellular environment. It is also plausible that the reduction of Smad4, a common target of miR-182 and miR-183,^42^^,^^43^ contribute to the vascular remodeling in HHT patients with *Drosha* mutations.

We found that the catalytic activity of Ago2 is necessary for processing hairpins in pri-miR-183 in the absence of FL-Drosha. Dicer-independent, Ago2-dependent cleavage of pre-miR-451 has been reported previously.^6^^,^^7^^,^^8^ Ago2 is less efficient than Dicer at cleaving pre-miR-451.^6^ We found that Ago2 is more efficient than Drosha in processing miR-183/96/182 hairpins, because (i) miR-183/96/182 levels are higher in ΔN cells than FL cells, and (ii) when Drosha is depleted in FL cells, miR-183/96/182 levels increase. Increase in miR-183 is reported in Wilms tumors, in which the catalytic activity of Drosha is inactivated due to mutations in the RNase III domains.^10^^,^^11^^,^^12^^,^^13^^,^^14^^,^^15^^,^^44^ Ago2 mutations have not been identified in Wilms tumors, however, we speculate that Ago2-dependent processing of miR-183/96/182 hairpins might facilitate the increase in miR-183 in Wilms tumors.

Although the majority of the miR-183 cluster miRNAs were identical to the reference sequence, ΔN cells also contained miR-183 and miR-96 isomiRs that were 1- or 2-nt longer at the 5′-end. This suggests that the Ago2-dependent cleavage site is less specific than the Drosha cleavage site and the addition of extra nucleotides at the 5′-end of isomiR-183 might alter the seed sequence, which result in the alternative recognition and/or silencing of target mRNAs in ΔN cells. The miR-183 cluster is abundantly expressed in the retina and plays an essential role in its development and homeostasis.^45^ Inactivation of an individual or multiple miRNAs in the miR-183 cluster in mice leads to retinal degeneration.^45^^,^^46^^,^^47^^,^^48^ It has been reported that Ago2 is present in both retinal neurons and glia,^26^ and depletion of Ago2 results in the reduction of miR-182 and miR-183.^49^ Furthermore, an HITS-CLIP assay using whole retina lysates find that the miR-183 cluster is the most abundant group of miRNAs bound to Ago2,^26^ which is consistent with our finding of the Ago2 role in pri-miR-183 processing. In the retina, depletion of Ago2 results in a reduction of miR-183 and -182 and retina degeneration,^49^ despite the presence of Drosha, suggesting that Ago2-mediated pri-miR-183 processing might be a predominant mechanism in the retina, similar to Ago2-dependent pre-miR-451 processing in erythrocytes.^6^ Increased expression of the miR-183 cluster is associated with various human disorders, including cancer, autoimmune diseases, and neuronal diseases.^25^ Furthermore, an increase in Ago2 protein amount along with post-translational modifications of Ago2, such as phosphorylation of tyrosine and serine residues and acetylation of lysine residues, is associated with poor prognosis and survival of cancer patients.^50^^,^^51^ Our study suggests that an increase in the protein stability, catalytic activity, or nuclear localization of Ago2 may underlie the increased expression of miR-183/96/182 in cancer.^25^

### Limitations of the study

This study has some potential limitations. The ΔN-Drosha cells were generated using HEK293T cells, which raises the possibility that the pri-miRNA processing activity of ΔN-Drosha, as well as the role of Ago2 in the processing of pri-miR-183, may be observations specific to this cell type. Further research is needed to elucidate the mechanism by which Drosha-NTR interacts with pri-miRNAs, particularly concerning the structure of Drosha-NTR. Lastly, further investigation is warranted to understand the mechanism by which Ago2 facilitates the processing of pri-miR-183, as well as the potential contribution of Ago2 in the deregulation of the miR-183 cluster of miRNAs in various human disorders.

## STAR★Methods

### Key resources table

**Table:** 

REAGENT or RESOURCE	SOURCE	IDENTIFIER
**Antibodies**		

Rabbit monoclonal anti-Argonaute 2	Cell Signaling Technology	Cat# 2897; RRID:AB_2096291
Mouse monoclonal anti-β-Actin	Sigma-Aldrich	Cat# A5441; RRID:AB_476744
Rabbit polyclonal anti-DDX5	Abcam	Cat# ab21696; RRID:AB_446484
Rabbit polyclonal anti-DGCR8	Proteintech	Cat# 10996-1-AP; RRID:AB_2090987
Rabbit polyclonal anti-Drosha	Bethyl	Cat# A301-886A; RRID:AB_1309798
Mouse monoclonal anti-Glyceraldehyde-3-Phosphate Dehydrogenase	Millipore	Cat# MAB374; RRID:AB_2107445
Rat monoclonal anti-GATA1	R and D Systems	Cat# MAB17791; RRID:AB_2108402
Rabbit polyclonal anti- Lamin A/C	Cell Signaling Technology	Cat# 2032; RRID:AB_2136278
Mouse monoclonal anti-Puromycin	Kerafast	Cat# EQ0001; RRID:AB_2620162
Rabbit polyclonal anti-RPL11	Proteintech	Cat# 16277-1-AP; RRID:AB_2181292
Mouse monoclonal anti-RPS19	Santa Cruz Biotechnology	Cat# sc-100836; RRID:AB_1129199
Rabbit polyclonal anti-RPS24	Abcam	Cat# ab102986; RRID:AB_10711571
Rabbit polyclonal anti-RPS26	Abcam	Cat# ab104050; RRID:AB_10710999
Rabbit polyclonal anti- RPSA (67kDa Laminin Receptor)	Abcam	Cat# ab137388; RRID:AB_2715562
Rabbit polyclonal anti-SMAD1	Thermo Fisher Scientific	Cat# 38-5400; RRID:AB_2533373
Rabbit polyclonal anti-phospho-Smad1 (Ser463/465)/ Smad5 (Ser463/465)/ Smad8 (Ser426/428)	Cell Signaling Technology	Cat# 9511; RRID:AB_331671
Goat polyclonal IRDye 680RD Goat anti-Rabbit IgG	LI-COR Biosciences	Cat# 926-68071; RRID:AB_10956166
Goat polyclonal IRDye 800CW Goat anti-Rabbit IgG	LI-COR Biosciences	Cat# 926-32211; RRID:AB_621843
Goat polyclonal IRDye 680RD Goat anti-Mouse IgG	LI-COR Biosciences	Cat# 926-68070; RRID:AB_10956588
Goat polyclonal IRDye 800CW Goat anti-Mouse IgG	LI-COR Biosciences	Cat# 926-32210; RRID:AB_621842
Goat polyclonal anti-rabbit IgG, HRP-linked	Cell Signaling Technology	Cat# 7074; RRID:AB_2099233
Horse polyclonal anti-mouse IgG, HRP-linked	Cell Signaling Technology	Cat# 7076; RRID:AB_330924
Goat polyclonal anti-rat IgG, HRP-linked	Cell Signaling Technology	Cat# 7077; RRID:AB_10694715

**Chemicals, peptides, and recombinant proteins**		

Puromycin	InvivoGen	Cat# ant-pr-1
DMEM-high glucose	HyClone lab	Cat# SH30022.01
Lipofectamine2000	Invitrogen	Cat# 11668-030
FCS	Hyclone	Cat# SH3007103
Lipofectamine RNAiMax	Invitrogen	Cat# 13778-150
SuperSignal™ West Dura extended duration substrate	ThermoFisher	Cat# 34076
Nitrocellulose blotting membrane	Genesee Scientific	Cat# 84-875
Polybrene	Sigma-Aldrich	Cat# TR-1003
Trypsin	Life technologies	Cat# 25200-072
Proteinase K	Invitrogen	Cat# P/N100005393
Protease Inhibitor	Sigma	Cat# P8340
Phosphatase Inhibitor	Sigma	Cat# P5726
ATTO 680	Jena bioscience	Cat# NU-821-680
RNase Inhibitor	Promega	Cat# N2111
RNase inhibitor	Invitrogen	Cat# AM2696
DNase I	Ambion	Cat# AM2238
SDS-PAGE sample buffer	Invitrogen	Cat# NP0007
SDS-PAGE reducing agent	Invitrogen	Cat# NP0009
Dynabeads Protein A	Invitrogen	Cat# 10002D
Dynabeads Protein A	Invitrogen	Cat# 10004D
MTT	Millipore	Cat# CT02

**Critical commercial assays**		

hsa-miR-183 Taqman assay	Applied Biosystems	Cat# 4427975-002269
has-miR-182 Taqman assay	Applied Biosystems	Cat# 4427975-002334
hsa-miR-96 Taqman assay	Applied Biosystems	Cat# 4427975-000186
hsa-miR-21 Taqman assay	Applied Biosystems	Cat# 4427975-000397
hsa-miR-103 Taqman assay	Applied Biosystems	Cat# 4427975-000439
hsa-miR-105 Taqman assay	Applied Biosystems	Cat# 4427975-002167
hsa-miR-199a Taqman assay	Applied Biosystems	Cat# 4427975-000498
hsa-miR-24 Taqman assay	Applied Biosystems	Cat# 4427975-000402
hsa-miR-34a Taqman assay	Applied Biosystems	Cat# 4427975-000426
hsa-miR-330 Taqman assay	Applied Biosystems	Cat# 4427975-002230
cDNA synthesis kit	Bio-Rad	Cat# 17088890
iQ SYBR Green Supermix	Bio-Rad	Cat# 1708882
RNeasy Mini kit	Qiagen	Cat# 74104
Riboprobe System-T7 Kit	Promega	Cat# P1440

**Deposited data**		

Small RNAseq Data	This paper	GSE229069

**Experimental models: Cell lines**		

HEK 293T Cell line	ATCC	Cat# CRL-3216
ΔN Drosha cells	Generated in-house	N/A
miR-183KO cells	Generated in-house	N/A

**Oligonucleotides**		

siAgo2	Sigma-Aldrich	Cat# SAHI-Hs02_00343736
siAgo2	ThermoFisher	Cat# n281589
siDGCR8	Sigma-Aldrich	Cat# SASI-Hs02_00355944
siDrosha	Dharmacon™	Cat# L-016996-00-0005
siControl	Dharmacon™	Cat# D-001206-13-05
PCR and qPCR Primer sequences please see Table S1	This paper	N/A

**Recombinant DNA**		

lentiCRISPR v2	Addgene	RRID:Addgene_52961
pMD2.G	Addgene	RRID:Addgene_12259
psPAX2	Addgene	RRID:Addgene_12260
pcDNA3.1(+)	Thermo Fisher	Cat# V79020

**Software and algorithms**		

GraphPad Prism	GraphPad	RRID:SCR_002798

### Resource availability

#### Lead contact

Further information and requests for resources and reagents should be directed to and will be fulfilled by the Lead Contact, Akiko Hata (akiko.hata@ucsf.edu).

#### Materials availability

This study did not generate new unique reagents.

### Experimental model and study participant details

#### Cell lines and culture conditions

Human embryonic kidney (HEK) 293T cells (ATCC, CRL-3216) were cultured in DMEM (high glucose, Sigma-Aldrich, SH30022.01) with 10% FCS (Hyclone, SH3007103), 1% penicillin/streptomycin at 37˚C and 5% CO_2_ under the protocol (#BU086922-05) approved by the UCSF Institutional Biosafety Committee (approval date 08/28/2023). Drosha ΔN-Drosha clones were maintained in the same media except containing 5 nM puromycin. For serum starvation, cells were cultured in DMEM with 1% FCS. To downregulate the *Drosha*, *Ago2*, and *Dgcr8* expression in FL-Drosha and ΔN-Drosha clones, siRNAs were introduced into clones using LipofectamineTM RNAiMAX transfection Reagent (Invitrogen, 13778-150).

### Method details

#### Generation of Drosha ΔN-Drosha cell lines by CRISPR-Cas9 genome editing

Two gRNAs were designed for each target within the human Drosha gene to facilitate genomic deletion of exon 5, using an online tool by Synthego. The sequences of the gRNAs are listed in the supplemental information. The gRNAs were cloned into the lentiCRISPR v2 plasmid (Addgene plasmid #52961). HEK293T cells were simultaneously transfected with PMD2.G (Addgene plasmid #12259) and psPAX2 (Addgene plasmid #12260) using Lipofectamine 2000 (Invitrogen, 11668030) to generate the lentivirus. Six hr after the transfection, the culture media was replaced with DMEM (high glucose, Sigma-Aldrich) supplemented with 10% FCS. After 48 hours, the supernatant was collected, filtered through a 0.45μm filter, and used to infect HEK293T cells with polybrene (8μg/ml, Sigma-Aldrich, TR-1003). The infected cells were selected in media containing puromycin (5 ng/μl), puromycin-resistant cells were trypsinized, and a single cell was sorted into 96-well plates by FACS (FACS AriaIII, BD Biosciences). After 30 days, clonal cell lines were expanded and subjected to genotyping. The genotyping primer sequences are listed in the supplemental Information.

#### Identification of Drosha ΔN-Drosha clones by genomic PCR

To distinguish the wild type Drosha alleles from the mutant (ΔN-Drosha) alleles, trypsinized cells were pelleted, resuspended in 10 mM Tris (pH 8.7), heated at 95°C for 10 min, incubated with proteinase K (0.5 μg/μl) for 20 min at 37°C, inactivated at 95°C for 15 min, and used as DNA template for genomic PCR analysis. The condition of genomic PCR is as follows: initial denaturing reaction at 95°C for 2 min; 35 cycles of denaturation at 95°C for 30 sec, annealing at 56°C for 30sec, and elongation at 72°C for 30 sec, followed by the final extension reaction at 72°C for 5 min. Primers designed to amplify the genomic region surrounding the site of deletion (primer #1-3) are listed in supplemental information. The wild type allele yields no PCR fragments by primer #1 and #2 but yield PCR fragments (621 bp) by primer #1 and #3. The exon 5-deleted (Δex5) allele yields PCR fragments (530 bp) by primer #1 and #2 but no fragments by primer #1 and #3.

#### SDS-PAGE and immunoblot analysis

Cell lysates were denatured in sample buffer (Invitrogen, NP0007) with reducing agent (Invitrogen, NP0009) for 5 min at 95°C, separated by SDS-gel electrophoresis, and transferred to nitrocellulose blotting membrane (Genesee Scientific). Membranes were blocked with TBST (20 mM Tris, 150 mM NaCl, pH 7.6, and 0.1% Tween20) with 3% nonfat dry milk for 1 hr and incubated with primary antibody in TBST with 1% milk overnight at 4°C. Chemiluminescence signals were detected using SuperSignal™ West Dura extended duration substrate (ThermoFisher, 34076) and imaged using an Odyssey Dlx Imaging System (LI-COR). Following antibodies were used for immunoblot: Anti-Drosha antibody (1:500 dilution, Bethyl, A301-866A), anti-Dgcr8 (1:500 dilution, Proteintech,10996-1-AP), anti-Ddx5 (1:200 dilution, Abcam, ab21696), anti-Ago2 (1:500 dilution, Cell signaling Technology, 2897), anti-GAPDH (1:5000 dilution, Millipore, MAB374), anti-Lamin A/C (1:2500 dilution, Cell signaling Technology, 2032), anti-Gata1 (1:200 dilution, R&D, MAB17791-SP), anti-puromycin (1:2000 dilution, Kerafast, 3RH11), anti-Rpl11 (1:300 dilution, Proteintech,16277-1-AP), anti-Rpsa (1:300 dilution, Abcam, ab137388), anti-Rps24 (1:300 dilution, Abcam, ab102986), anti-Rps26 (1:300 dilution, Abcam, ab104050), anti-Rps19 (1:300 dilution, Santa Cruz Biotechnology, sc-100836), anti-Smad1 (1:500 dilution, Invitrogen, 38-5400), phospho-Smad1/5/8 (1:100 dilution, Cell signaling Technology, 9511), anti-β-actin (1:5000 dilution, Sigma-Aldrich, A5441), IRDye 680RD goat anti-rabbit IgG (H + L) (LI-COR, 926-68071), IRDye 800CW goat anti-rabbit IgG (H + L) (LI-COR, 926-32211), IRDye 680RD goat anti-mouse IgG (H + L) (LI-COR, 926-68070), and IRDye 800CW goat anti-mouse IgG (H + L) (LI-COR, 926-32210). anti-Rabbit-IgG-HRP-linked (1:3000 dilution, Cell signaling Technology, 7074), anti-Mouse-IgG-HRP-linked (1:3000 dilution, Cell signaling Technology, 7076),anti-Rabbit-IgG-HRP-linked (1:3000 dilution, Cell signaling Technology, 7077).

#### Ago2 expression plasmids

Wild type Ago2 expression construct was reported previously.^52^ The coding sequence of Ago2-D597A mutant was PCR amplified from the lentiviral vector carrying Ago2-D597A^53^ and subcloned into the pcDNA3.1(+) vector at HindIII and EcoRI restriction sites.

#### Quantitative reverse transcriptase-polymerase chain reaction (qRT-PCR) analysis

Total RNAs were extracted by RNeasy Mini Kit (#74104, Qiagen) and subjected to generate cDNAs by RT reaction (#17088890, Bio-Rad). qPCR was performed using iQ SYBR Green Supermix (#1708882, Bio-Rad). All reactions were run in triplicates. The relative expression values were determined by normalization to *GAPDH* transcript levels and calculated using the ΔΔCT method.

Primers used for qRT-PCR are listed in supplemental information.

#### Quantitative miRNA analysis

Mature miRNAs were determined using TaqMan microRNA Assays (Applied Biosystems Inc.). Normalization was performed with the small nuclear RNA U6 (RNU6B; Applied Biosystems Inc.). All real-time reactions, including no-template controls and real-time minus controls, were run using the CFX Connect Real-Time PCR System (Bio-Rad) and performed in triplicate. Relative expression was calculated using the ΔΔCT method.

#### Immunoprecipitation assay

Cells were lysed in SBB buffer (1% Triton X-100, 150mM NaCl, 50mM Tris-Cl at pH 7.5, 1mM EDTA) supplemented with protease inhibitors (Sigma, P8340) and phosphatase inhibitor (Sigma, P5726). Cell lysates were incubated at 4°C for 30 min and centrifuged at 12,000 g for 10 min at 4°C. Lysates were incubated with anti-Drosha, anti-Ago2, anti-Dgcr8, and anti-IgG (negative control) nutating overnight at 4°C followed by the addition of Dynabeads™ Protein A/G (Invitrogen, 10002D/10004D) and rocking for 4 hr at 4°C. The magnetic beads were precipitated and rinsed with SBB buffer for 5 min at 4°C for three times, followed by adding sample buffer (Invitrogen, NP0007) with reducing agent (Invitrogen, NP0009) and heated at 95°C for 3 min.

#### Nuclear and cytoplasmic fractionation

Cells were washed with PBS twice, scrape off and pelleted by centrifuging at 4,500 g for 5 min. Cells were then swelled by adding 5 volume of lysis buffer (10 mM HEPES, pH 7.9, with 1.5 mM MgCl_2_, 10 mM KCl, 1mM DTT and protease inhibitor, sigma, P8340) and homogenized. After centrifugation at 10,000 g for 15 min, the supernatant was collected as a cytoplasmic fraction. The crude nuclei pellet was resuspended in 2/3 volume extraction buffer (20 mM HEPES, pH 7.9, with 1.5 mM MgCl_2_, 0.42 M NaCl, 0.2 mM EDTA, 25% (v/v) Glycerol, 1mM DTT and protease inhibitor sigma P8340) and homogenized with a tissue homogenizer. After centrifuging at 20,000 g for 5 min, the supernatant was collected as a nuclear fraction.

#### Next generation RNA-seq and small RNA-seq and analysis

Total RNAs were extracted from cells using TRIzol (Invitrogen). The quality of RNAs was evaluated by 2100 Bioanalyzer Instrument (Agilent Technologies) and the samples with RIN>8.0 were sent to Beijing Genome Institute (BGI) for RNA sequencing and small RNA sequencing. The sequencing was performed with DNBSEQ™ technology platforms. Adapter removed clean data were generated by BGI. Quality control, index generation and mapping for RNA sequencing were done with Salmon software tool. Differential gene expression was analyzed with R package DESeq2_1.4.5. Quality control for small RNA sequencing data were done by fast quality filter and fastx trimmer.

FASTQ sequences were aligned to the human reference genome (GRCh38) by miRDeep2. The reads were aligned against known miRNAs from miRBase (version 19.0). To process paired-end sequencing, reads were aligned separately covering the mature miRNA both on the forward and reverse read and the obtained number of matches were averaged. The match counts were normalized by a linear scaling method: Trimmed Mean of M-values (TMM) and tested for differential expression using the edgeR package with default settings. To calculate the fold change (FC) of miRNA levels in +/+ cells and Δex5/Δex5 cells, normalized miRNA counts in +/+ cells (n=4) or ex5/Δex5 cells (n=5) were averaged, and the FC of miRNAs was calculated. To analyze the change in proportion of trimmed or added reads at each ends of miRNAs listed in Table 1, we calculated the number of miRNAs reads with the size shorter or longer than the reference sequence as reads per million (RPM), followed by calculating the ratio of RPM in Δex5/Δex5 cells: +/+ cells.

#### Chromatin immunoprecipitation (ChIP) assay

Cells were crosslinked treated with 1% Formaldehyde for 15 min at room temperature Followed by quenching with 1M Glycine, cells were washed with PBS and lysed with lysis buffer (50 mM Tris-Cl pH 8.1, 10 mM EDTA, 1% SDS and protease inhibitors). Genomic DNAs were sheared to average length of 200-500bp by sonication, followed by clearing lysates by centrifugation at 12,000g for 10 min at 4°C. Incubate the supernatant with protein A/G dynabeads (invitrogen 10002D/10004D) for 1 hr at 4°C, dilute the pre-cleared sample to 1:10 ration with dilution buffer (20 mM Tris-Cl pH 8.1, 150 mM NaCl, 2 mM EDTA, 1% Triton X-100 and protease inhibitor) and 1/10 volume was kept as input before incubation with anti-Drosha overnight at 4°C followed by 1 hr incubation with protein A/G dynabeads at 4°C. After the dynabeads were washed with a buffer I (20 mM Tris-Cl pH 8.1, 150 mM NaCl, 2 mM EDTA, 1% Triton X-100, 0.1% SDS), buffer II (20 mM Tris-Cl pH 8.1, 500 mM NaCl, 2 mM EDTA, 1% Triton X-100, 0.1% SDS), and buffer III (10mM Tris-Cl pH8.1, 250mM LiCl, 1mM EDTA, 1%NP-40, 1% Deoxycholate) at 4°C, the dynabeads were further washed twice with cold TE (10 mM Tris-Cl pH 8.1, 1 mM EDTA). The dynabeads were incubated in 250 μl elution buffer (200 mM NaHCO_3_, 1% SDS) at room temperature for 15 min twice. The eluates were mixed with 1/25 volume 5M NaCl and incubated at 65°C for 4 h. 1/50 volume of 0.5 M EDTA, 1/25 volume of Tris-Cl pH 6.5, 1/100 volume of proteinase K (10 mg/ml) were added and incubated at 45°C for 1 hr. Precipitated genome fragments were purified with QIAquick PCR Purification Kit, followed by PCR analysis. Primers used for ChIP assay are listed in supplemental information.

#### Proliferation assay

Cell growth was monitored by cell counting and 3-(4,5-dimethylthiazol-2-yl)-2,5-diphenyltetrazolium bromide (MTT) assay using MTT cell growth assay kit (#CT02, Millipore). FL or ΔN-Drosha cells (1x10^5^) were seeded in 12-well plates and cultured in DMEM containing 10% or 1% FCS. 8, 16, 24, 32, or 40 hr after the media change, cells were harvested and counted by a hemocytometer. For MTT assay, MTT dye was added to each well, incubated at 37°C for 1 hr, followed by the addition of 0.1 mL isopropanol with 0.04 N HCl. The absorbance was measured at a wavelength of 570 nm.

#### Puromycin incorporation assay

Cells were cultured in the growth media (DMEM with 10% FCS) or starvation media (DMEM with 1% FCS) for 6 or 16 hr, followed by the treatment with 1μM puromycin at 37°C for 10 min. Total cell lysates were generated and subjected to SDS-PAGE and immunoblot with an anti-puromycin antibody (Kerafast, EQ0001).

#### *In vitro* pri-miRNA processing (IVP) assay

A partial pri-let7b (431-nt) or pri-miR-183 (432-nt) sequence was amplified from human genomic DNAs and used as *in vitro* transcription template in the reaction using Riboprobe System-T7 Kit (P1440, Promega) with a fluorescence dye (ATTO 680)-conjugated UTP (Aminoallyl-UTP-ATTO-680, NU-821-680, Jena Bioscience). FL and ΔN cells (∼3x10^6^ cells) were harvested in 300μl sonication buffer (20 mM Tris-HCL pH=8.0, 100 mM KCL, 0.2 mM EDTA RNase-free) and sonicated with 20% intensity for 5 sec for 3 times. After the sonication, cell lysates were subjected to the centrifugation (12,000 rpm at 4 °C for 15 mins). The fluorescein-labelled pri-let-7b or pri-miR-183 (0.1 μg) was mixed with the supernatant (total protein amount of 20-30 μg) supplemented with 6.4 mM MgCl_2_ and 0.5 U/μl Recombinant RNase Inhibitor (Promega) in total volume of 30 μl. After the incubation at 37°C for 45 min, the reaction mixtures were separated on a 15% Urea-PAGE gel at 90 V for 150 min to separate the processing product [pre-let7b (82-nt) or pre-miR-183 (110-nt)] from the substrate (pri-let7b or pri-miR-183). The gel image was captured by Odyssey Dlx Imaging System (LI-COR) and the amounts of pri- and pre-let7b and miR-183 were quantitated. The relative processing activity was calculated by the amount of pre-miRNA divided by the sum of pri-miRNA and pre-miRNA after being normalized by the Drosha protein amount. The low range single stranded RNA ladder (#N0364N, NEB) was stained with SYBR Gold (#S11494, Thermo Fisher Scientific) according to the manufacturer’s protocol and used as a molecular marker.

#### RNA immunoprecipitation (RIP) assay

HEK293T cells with FL and ΔN-Drosha Drosha were subjected to crosslinking with 1% formaldehyde for 15 min at room temperature. Nuclei were isolated and disrupted by sonication using Bioruptor (Diagenode). The sonicated lysates were cleared and subjected to immunoprecipitation with antibodies against RNA binding protein (RBP), such as Drosha, Ago2, or Dgcr8. After immunoprecipitation of RBP associated with RNAs, washing and elution, the pellets were subjected to 10 U DNase I treatment for 30 min at 37°C to remove any remaining DNA. Next, total RNAs were extracted using Trizol/phenol:chloroform (5:1), precipitated with ethanol, and dissolved in 20ul of nuclease free water. 5ul of RNA was used for 20ul cDNA synthesis reaction. qRT-PCR reactions were performed using pre-miR-primers by real-time PCR machine (CFX96, BioRad). 100 U/ml RNase inhibitor (SUPERase·in™) was used throughout the experiment. Immunoblot analysis was performed to quantitate the amount of RBP in the precipitates. To calculate the fold enrichment of each RIP reaction from qPCR data, first normalize the Ct value of the target RNA to the Ct of GAPDH (control) mRNA. The data are displayed as ‘fold enrichment’ of RNAs in RBP IP relative to IgG IP after the normalization of the amount of RBP in the IP sample.

#### Generation of miR-183 knock out cells

HEK293T cells with biallelic deletion in the miR-183 hairpin (183KO cells) were generated by CRISPR-Cas9 gene editing using following guide RNAs (gRNAs): gRNA1(F): 5′-GACCGTAGCAGCCGCTGCTG AGG-3′ and gRNA2(R): 5′-AAGTGGGTAAGGTGCTCCGG AGG-3′. HEK293T cells were transfected with gRNAs and Cas9-expressing clones using electroporation (1,200V, 30ms, 2 pulse). Cells were isolated by adding puromycin (1 μg/ml) to culture media (90%DMEM with 10% FCS), followed by isolating clones by limited dilution in 96-well plate. Clones were subjected to PCR analysis of genomic DNA (gDNA) (95˙C 15 sec, 60 ˙C 15 sec, 72 ˙C 15 sec, 30 cycles) using primers: F1:5′-CTGCTTGCCTCTCCGAGCCA-3′, F2: 5′-GTCAGTGAATTACCGAAGGGCC-3′, and R1:5′-CCAGGCAGTGTAAGGCGATCTG-3′. The wild type (WT) allele generates PCR product of 859-bp by F1/R1 and 422-bp by F2/R1. Two different 183KO alleles (KO1 and KO2) were validated by a shorter length of the PCR product 635-bp and 646-bp by F1/R1 in 183KO1 and 183KO2, respectively, and no PCR product was detected by F2/R1. Deletion of 224-bp and 213-bp in 183KO1 and 183KO2 cells were confirmed by sequencing, respectively.

### Quantification and statistical analysis

Graphs were generated with GraphPad PRISM software. Statistical significance was calculated in R version 3.2.3 by Student’s t test. The null hypothesis of the medians/means being equal was rejected at α = 0.05 and p values were generated by unpaired Student’s t test and presented in figures. The sample size was estimated by power analysis and is presented in the figure legend. All experiments were performed at least three times with biological triplicates each time.

## Data Availability

Sequencing data presented in this study will be available in the NCBI Gene Expression Omnibus (GEO) database (https://www.ncbi.nlm.nih.gov/geo/) under accession code: GSE229069. We did not generate any original code. Any additional information required to reanalyze the data reported in this work paper is available from lead contact upon request.
