# Supplementary material for: Essential role of the amino-terminal region of Drosha for the Microprocessor function
Source: iScience. 2023 Sep 20;26(10):107971. doi: 10.1016/j.isci.2023.107971 (PMC10558778; doi:10.1016/j.isci.2023.107971)
Supplement: Document S1. Figures S1–S13 and Tables S1–S6 [file mmc1.pdf]

iScience, Volume *26*

Supplemental information

Essential role of the amino-terminal region of Drosha for the Microprocessor function

Amit Prabhakar, Song Hu, Jin Tang, Prajakta Ghatpande, Giorgio Lagna, Xuan Jiang, and Akiko Hata

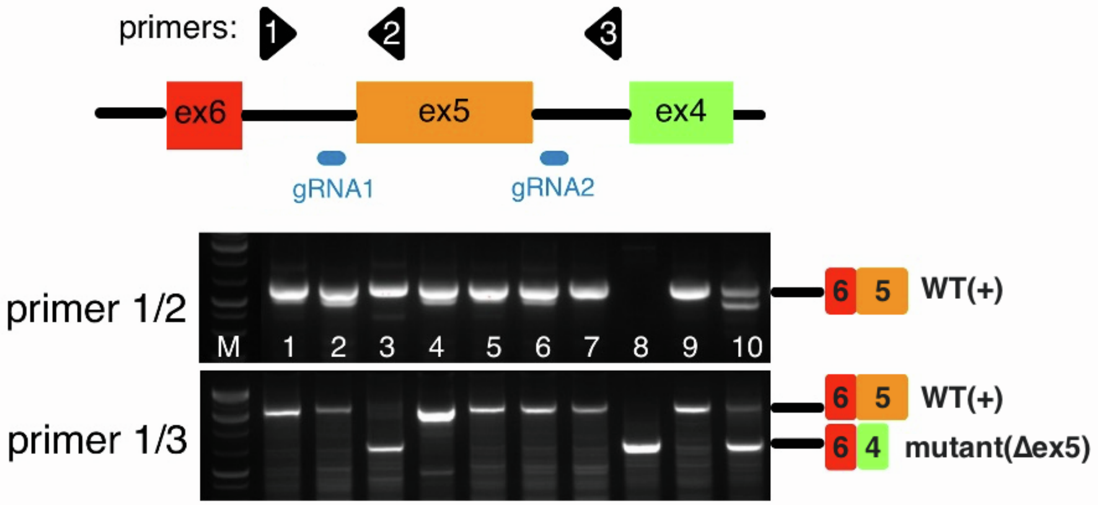

Figure S1 Identification of *Drosha* mutant clones in HEK293T cells, related to Figure 1. Two guideRNAs (gRNA1 and gRNA2, blue) were used to delete exon5 (Δex5) of *Drosha* by CRISPR/Cas9-based genome editing are indicated. Lanes 1-10 correspond to the genomic DNA from clones 1-10. Primers 1-3 (top) were used for genomic DNA analysis to distinguish wild type (WT; +) vs mutant (Δex5) allele of the Drosha gene. Results of PCR analyses of genomic DNA by 2 set of primers are shown (top: primers #1 and #2 and bottom: primers #1 and #3). M: molecular marker.

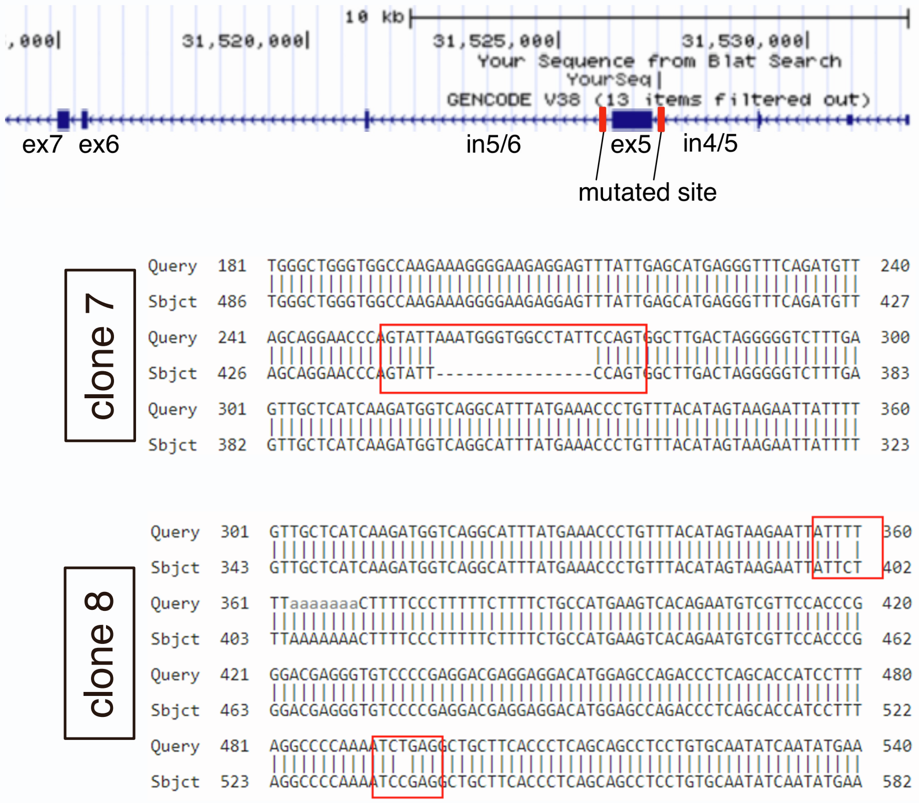

Figure S2 Mutations upstream of exon5 of the Drosha gene were identified in clone 7 and 8, related to Figure 1. Two gRNAs were used to delete a whole exon 5 (ex5) of human *Drosha* by CRISPR/Cas9-based genome editing (top). Mutated sequence in intron 4/5 in clone 7 (Δex5/+) and clone 8 (Δex5/Δex5) are shown (bottom).

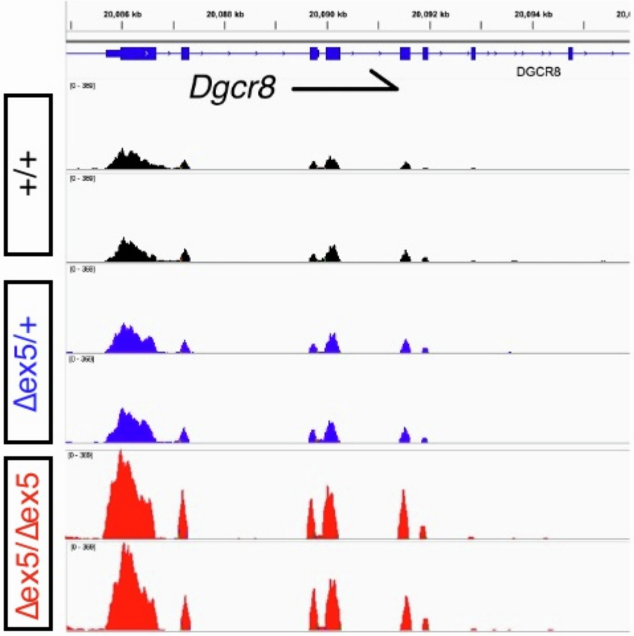

Figure S3 RNAseq analysis of the Dgcr8 transcripts in clone 4 (+/+), 7 (Δex5/+), and 8 (Δex5/Δex5), related to Figure 1. RNA-seq analysis confirms an increased level of reads corresponding to the Dgcr8 mRNA in Δex5/Δex5 cells (clone 8, red) in comparison with +/+ cells (clone 4, black) and Δex5/+ cells (clone 7, blue). Two libraries were generated from each clone and both RNA-seq data are shown.

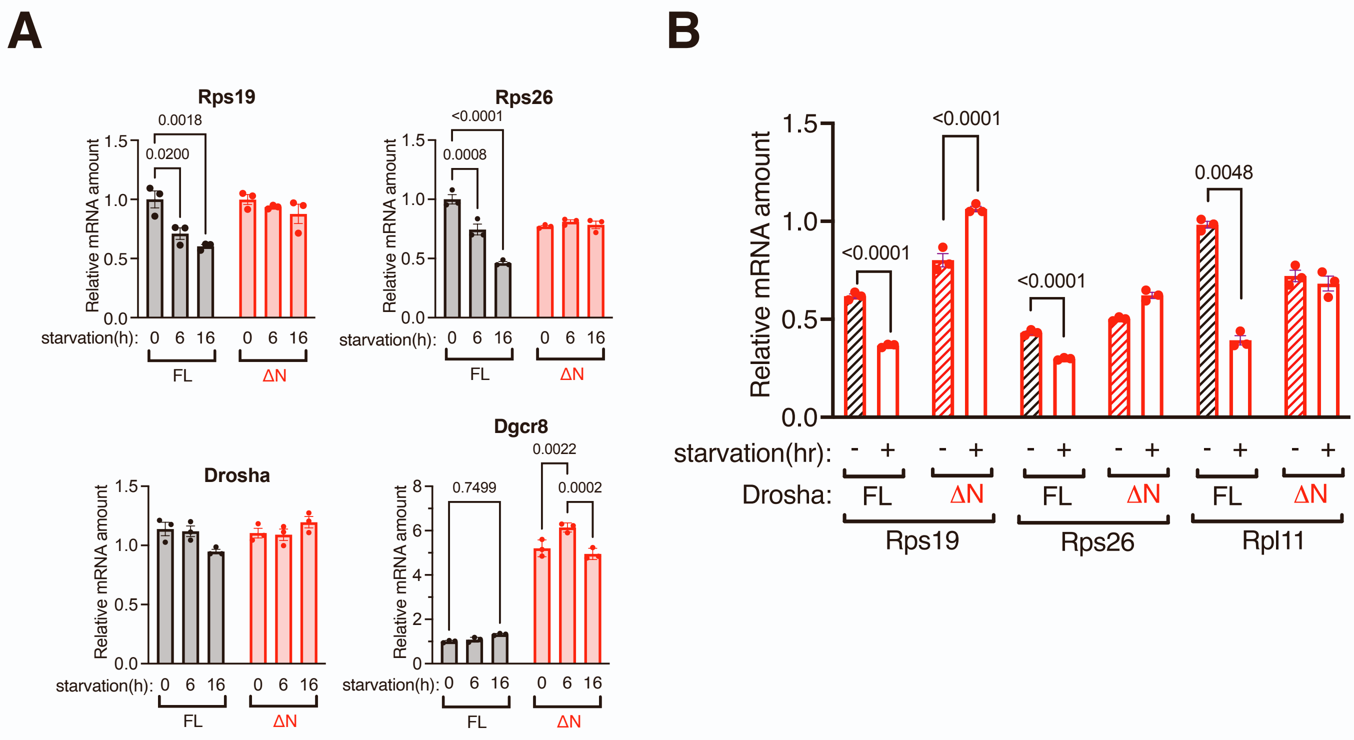

Figure S4 ΔN-Drosha is unable to control the levels of RPG mRNAs upon serum starvation, related to Figure 3. A. qRT-PCR analysis of the Rps19, Rps29, Drosha and Dgcr8 mRNAs relative to GAPDH mRNAs in FL (+/+) cells (black) and ΔN-Drosha (Δex5/Δex5) cells (red). Results are plotted as mean± SEM. n=3 independent experiments. **B.** ΔN-Drosha (Δex5/Δex5) cells, in which either FL-Drosha (FL) or ΔN-Drosha (ΔN) was exogenously expressed, were treated with or without serum starvation (1% serum) for 16 hr, followed by qRT-PCR of the Rps19, Rps26,and Rpl11 in . Results are plotted as mean± SEM. n=3 independent experiments.

Figure S5 Prabhakar et al.

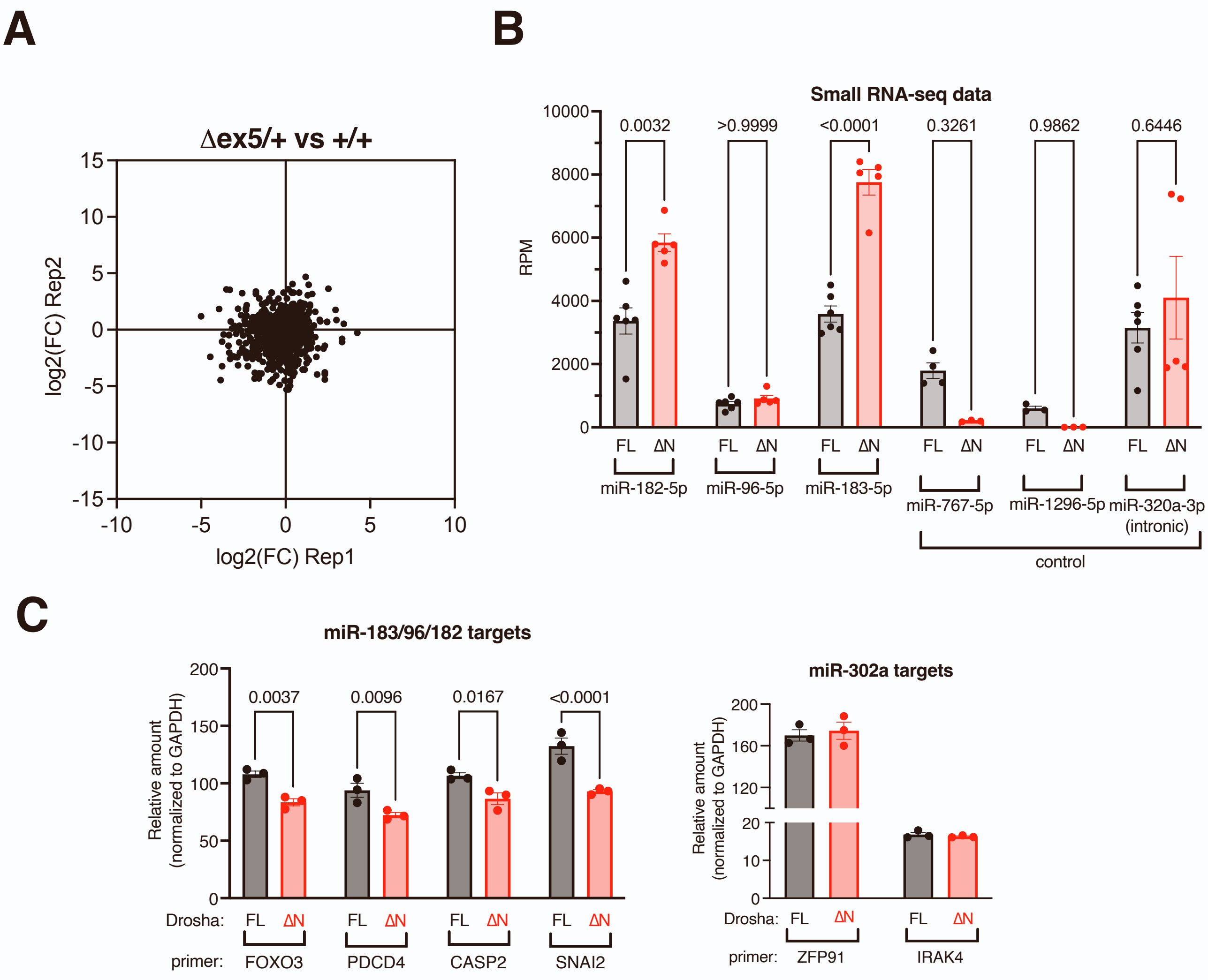

Figure S5 Higher amount of miR-183 cluster in ΔN-Drosha cells compared to FL-Drosha cells, related to Figure 4. A. Small RNA-seq analysis of miRNAs in +/+ cells (clone 4) and Δex5/+ cells (clone 7). The proportion of miRNA reads in the small RNA sequencing libraries from +/+ cells and Δex5/+ cells are compared and the fold change (FC) of miRNAs (log_2FC) are shown in scatter plot. Two libraries from two independent samples were generated from each clone. **B.** Small RNA-seq data of FL cells and ΔN-Drosha cells indicates similar level of expression of miR-182-5p, miR-96-5p and miR-183-5p in clones 4 and 8 despite miR-767-5p and miR-1296-5p (control) were significantly less in clone 8. A similar level of intronic miR-320a-3p was detected in FL and ΔN cells. Results are plotted as mean RPM (read per million reads)± SEM. n=6 for FL cells and n=5 for ΔN-Drosha cells. **C.** The levels of mRNAs of miR-183/96/182 targets (left) and miR-320a targets (right) in ΔN cells and FL cells were examined by qRT-PCR and plotted as mean± SEM. n=3.

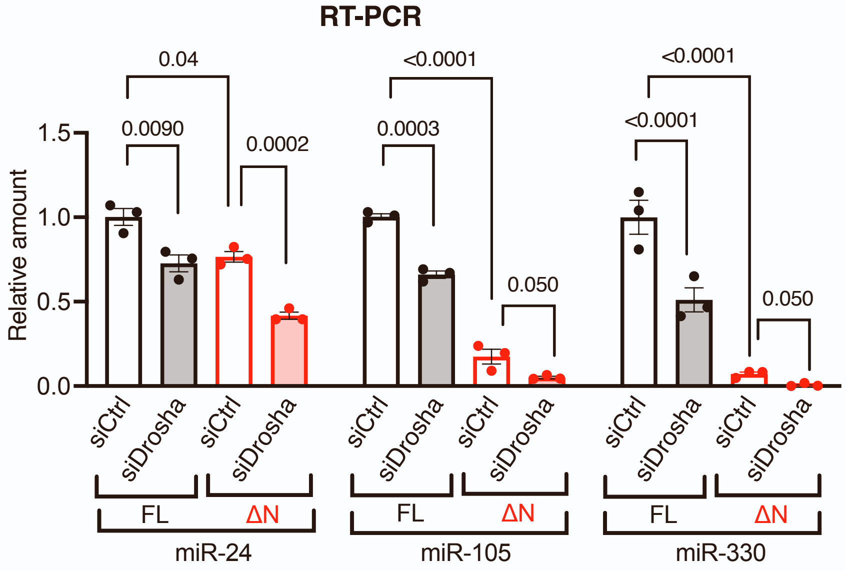

Figure S6 Drosha-dependent processing of miR-24, -105 and -330, related to Figure 4. FL or ΔN-Drosha cells transfected with siRNA against Drosha (siDrosha) or non-specific control siRNA (siCtrl) were subjected to qRT-PCR analysis of miR-24, -105, and -330. Relative amount of miRNAs normalized to U6 snRNA is plotted as mean± SEM. n=3.

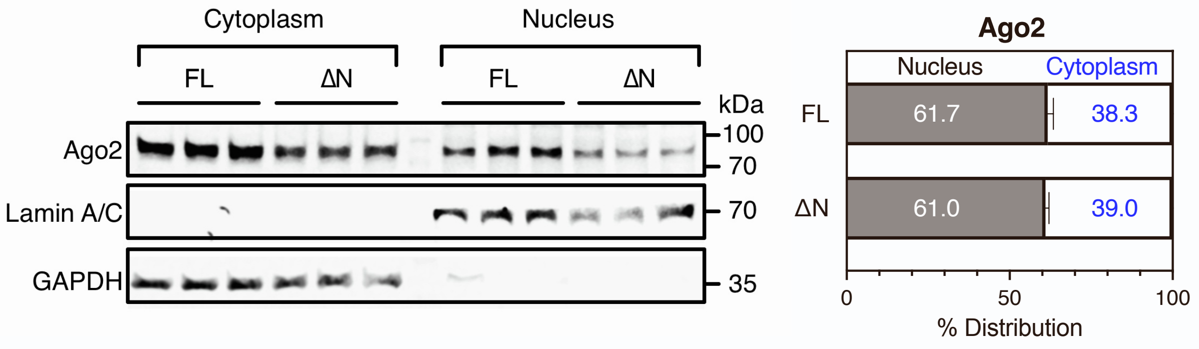

Figure S7 Ago2 localizes not only in the cytoplasm but in the nucleus, related to Figure 4. Nuclear and cytoplasmic fraction of FL and ΔN-Drosha cells were subjected to immunoblot analysis of Ago2, Lamin A/C (control for the nucleus), and GAPDH (control for the cytoplasm) (left). Relative distribution (%) of FL and ΔN-Drosha in the nucleus vs cytoplasm is shown (right).

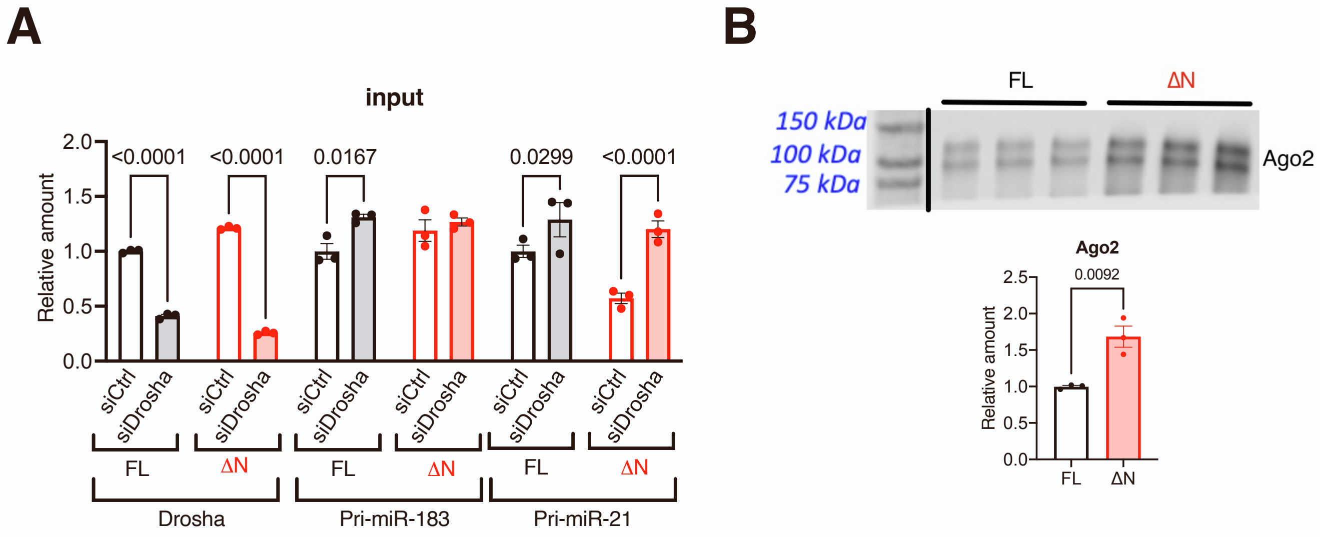

Figure S8 Similar amount of Ago2 protein was precipitated by anti-Ago2 antibody, related to Figure 4. A. The relative amount of Drosha, pri-miR-183, and pri-miR-21 mRNAs (normalized to GAPDH) in the input samples of of RIP assay (anti-Drosha IP) in FL and ΔN-Drosha cells transfected with siCtrl or siDrosha is plotted as mean± SEM. n=3 **B.** IP with anti-Ago2 antibody (αAgo2) but not nonspecific IgG (IgG) pulls down Ago2 protein together with associating RNAs in FL and ΔN-Drosha cells (top). The amount of Ago2 was quantitated and plotted as mean± SEM (bottom). n=3 independent experiments.

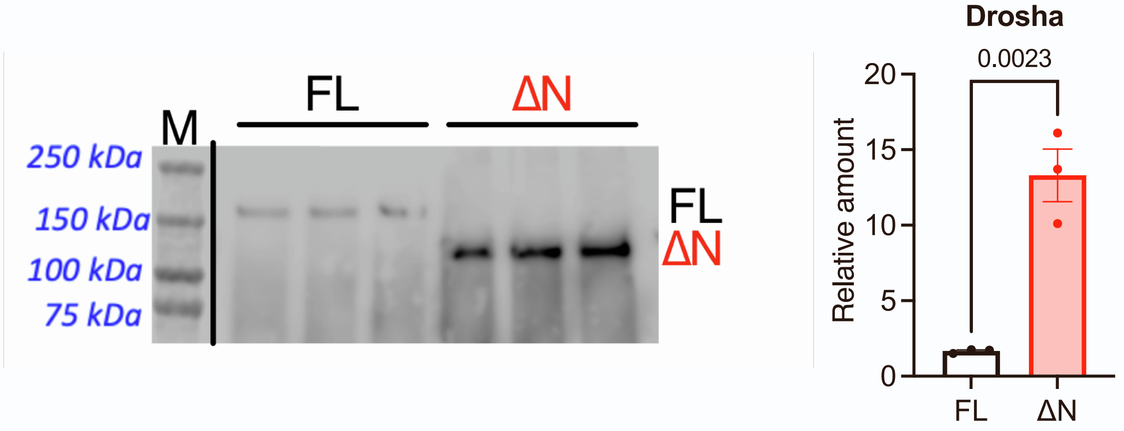

Figure S9 The amount of Drosha (FL or ΔN) in the IP samples of RIP after anti-Drosha IP in FL or ΔN-Drosha cells, related to Figure 4. RIP assay to access the interaction between Drosha and pri-miRNAs in FL and ΔN-Drosha cells. IP with anti-Drosha antibody was subjected to immunoblot with Drosha antibody (left). The amount of Drosha was quantitated and plotted as mean± SEM (right). n=3 independent experiments. M: molecular marker.

Figure S10 Prabhakar et al.

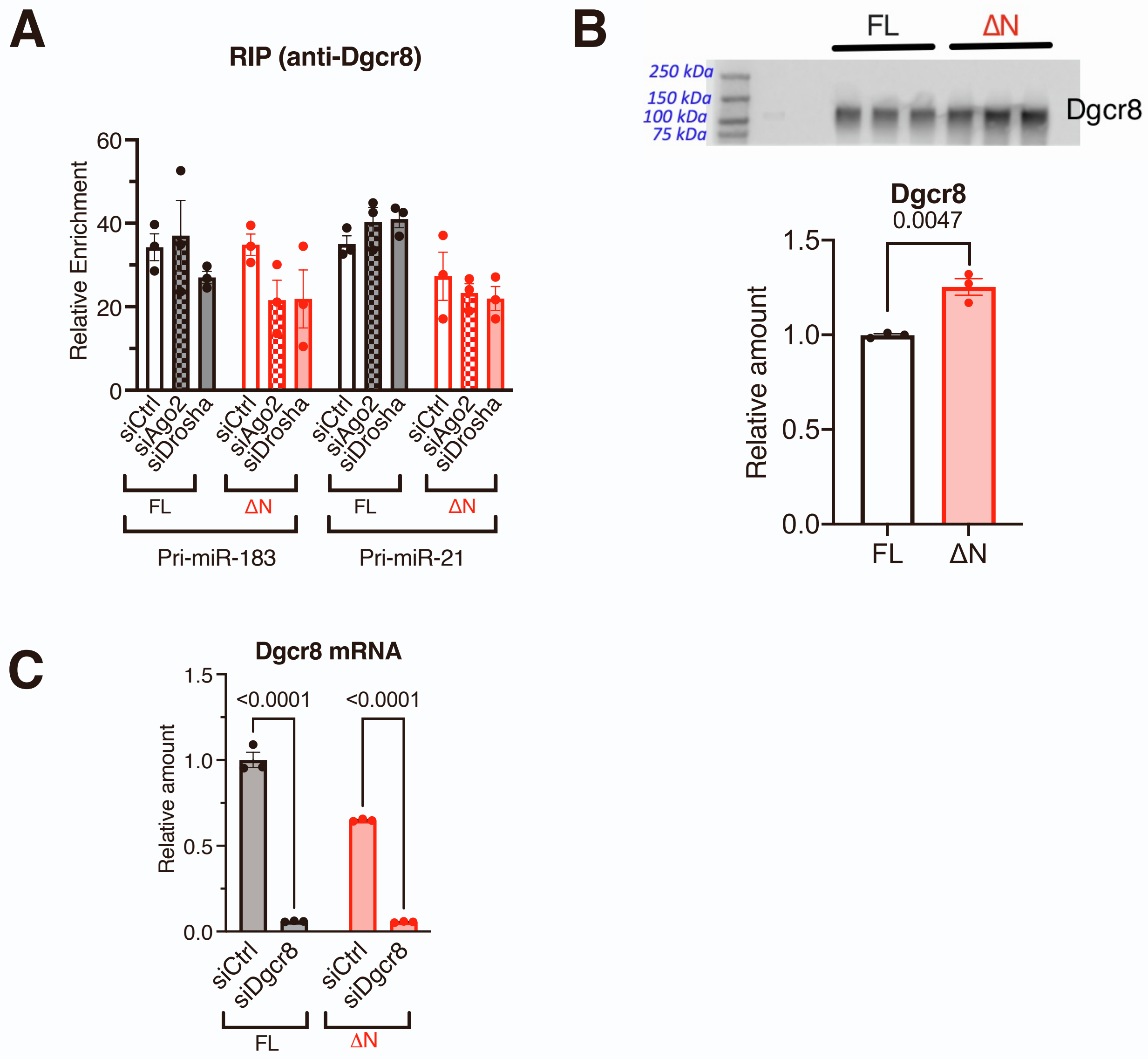

Figure S10 Association of Dgcr8 with pri-miR-183 or pri-miR-21 is not affected by the depletion of Ago2 or Drosha, related to Figure 4. A. RIP assay to access the interaction between Dgcr8 and pri-miR-183 or pri-miR-21 in FL and ΔN-Drosha cells transfected with siCtrl, siAgo2, or siDrosha. The amount of pri-miR-183 or pri-miR21 in the IP samples of anti-Dgcr8 antibody or non-specific IgG (control) was quantitated by qRT-PCR in triplicates. Relative enrichment Dgcr8 IP over control IgG IP was plotted as mean± SEM. n=3 independent experiments. **B.** IP with anti-Dgcr8 antibody was subjected to immunoblot with anti-Dgcr8 antibody (top). The amount of Dgcr8 was quantitated and plotted as mean± SEM (bottom). n=3 independent experiments. **c**. The relative amount of Dgcr8 mRNA (normalized to GAPDH) in the input samples of of RIP assay (anti-Ago2 IP) in FL and ΔN-Drosha cells transfected with siCtrl or siDgcr8 is plotted as mean± SEM. n=3

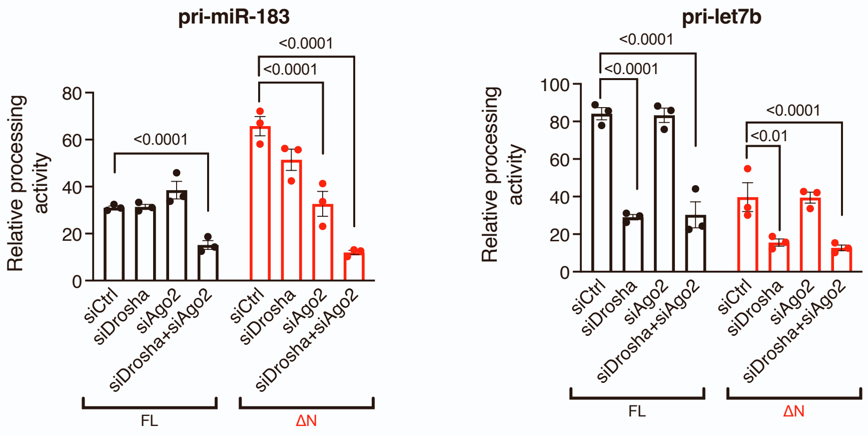

Figure S11 Processing of pri-miR-183 hairpins in ΔN cells requires Ago2, related to Figure 4. The In Vitro Processing (IVP) assay was performed by incubating IR-labeled pri-miR-183 (on the left) or pri-let7b (on the right) with whole cell lysates from FL or ΔN cells that had been transfected with siCtrl, siDrosha, siAgo2, or a combination of siDrosha and siAgo2. Following the reaction, pre-miR-183 and pre-let7b were separated from pri-miR-183 and pri-let7b using PAGE. Relative processing activities were calculated and are displayed as mean±SEM. n=3 independent experiments.

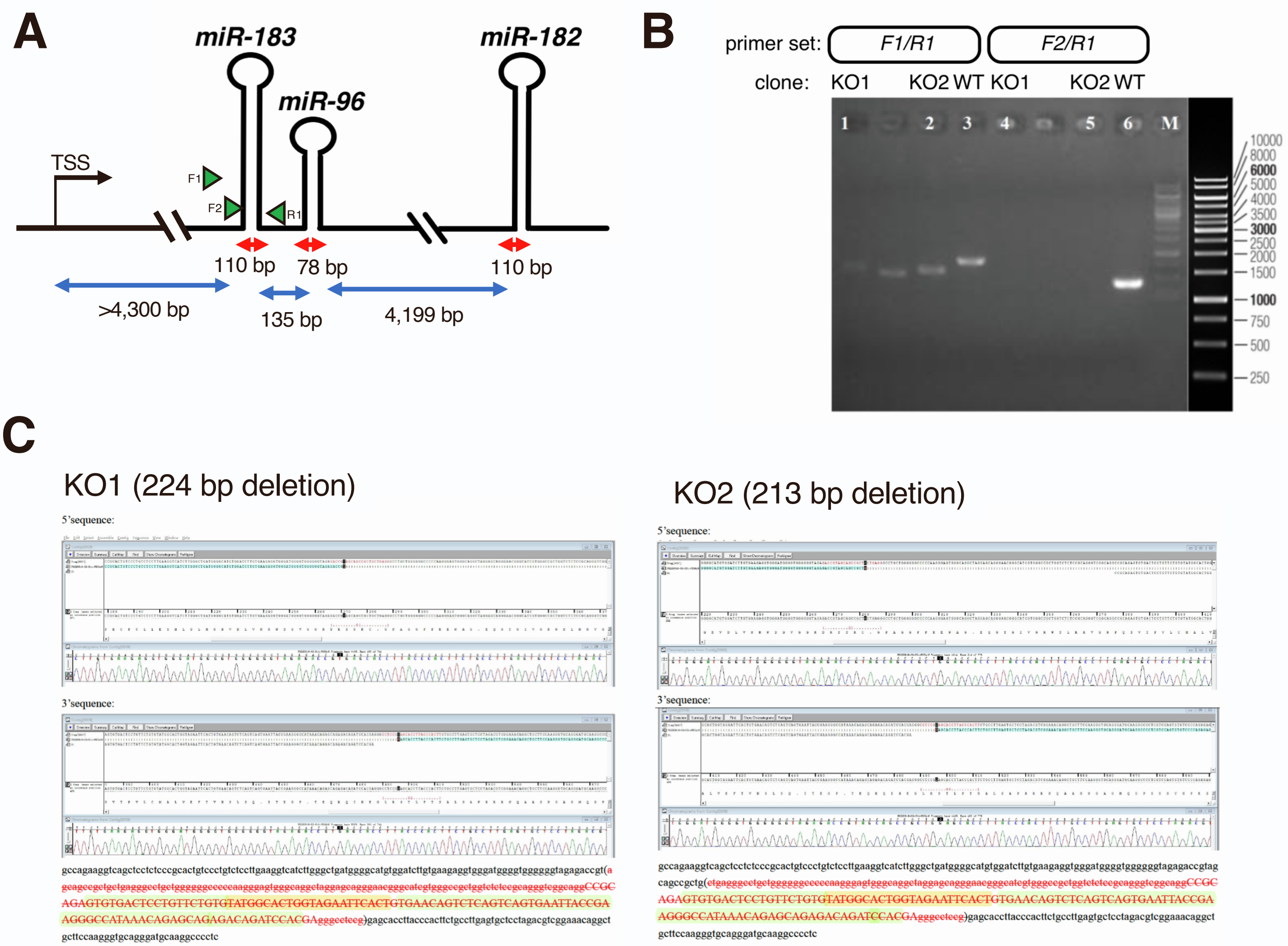

Figure S12 Generation of miR-183 knockout HEK293T cells by CRISPR/Cas9, related to Figure 5. **A.** Genetic architecture of miR-183 cluster on the chromosome 7 in human. The length of miR-183, -96, and -183 hairpins is shown in red. The distance between two miRNA hairpins is shown in blue. The black arrow indicates a transcription start site of the pri-miR-183/96/182. Green arrowheads indicate F1, F2, and R1 primers used in the genomic PCR analysis shown in B. TSS: transcription start site. **B.** Genomic PCR analysis validates the deletion of miR-183 hairpin in KO1 and KO2 cells. The wild type allele (WT) generates PCR product of 859-bp with F1/R1 (lane 3) and 422-bp with F2/R1 (lane 6). Two KO alleles (KO1 and KO2) generate PCR product of 635-bp (lane 1) and 646-bp (lane 2) with F1/R1, respectively, and no product with F2/R1 (lanes 4 and 5). **C.** Sequencing of genomic DNA confirms 224 bp and 213 bp deletion in KO1 (left) and KO2 cells (right) are indicated with overline. Green shade: miR-183 hairpin sequence. Orange shade: mature miR-183 sequence.

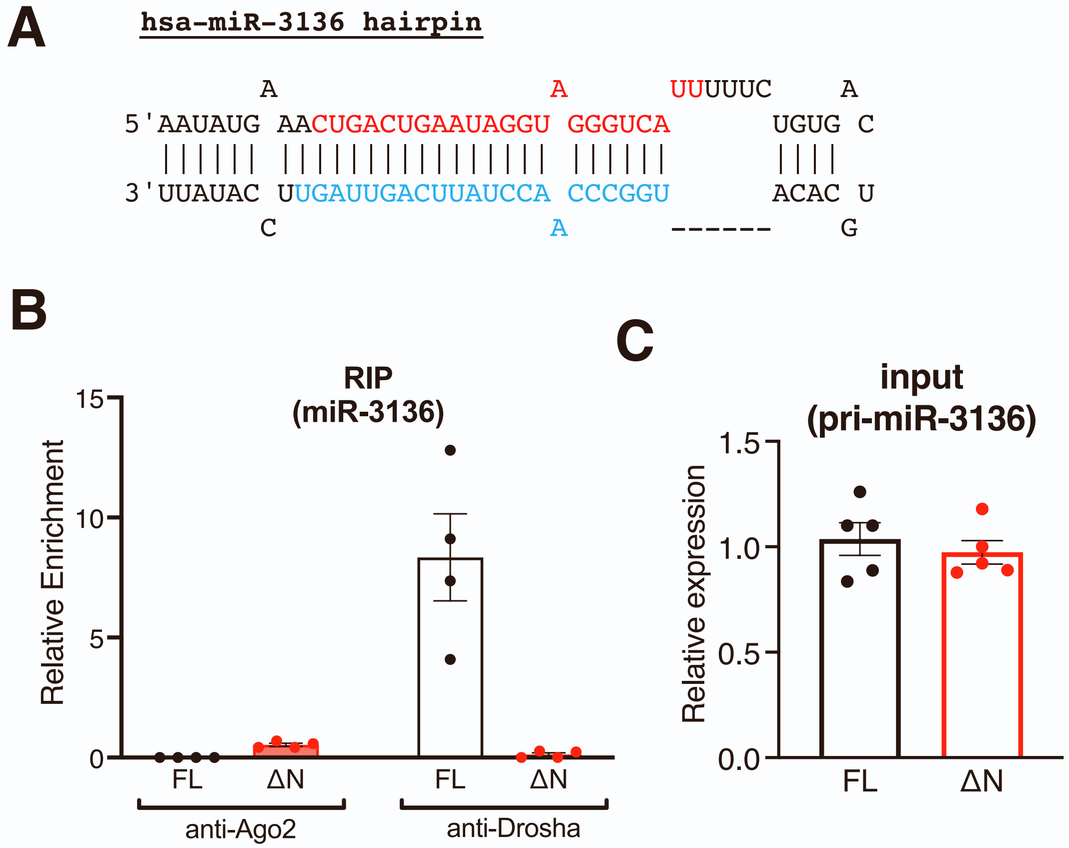

Figure S13 Ago2 does not associate with miR-3136 hairpin with 40-nt long stem, related to Figure 4. A. The hairpin structure of miR-3136 includes 40-nt stem with long mismatched nucleotides. Red: miR-3136-5p, Blue: miR-3136-3p. **B.** RIP assay (anti-Ago2 or anti-Drosha IP) was performed in FL and ΔN-Drosha cells. The amount of pri-miR-3136 in the immunoprecipitates of anti-Ago2 or anti-Drosha antibody or non-specific IgG (control) was quantitated by qRT-PCR. Relative enrichment Ago2 or Drosha IP over control IgG IP was plotted as mean± SEM. **C.** Relative amount of pri-miR-3136 mRNA (normalized to GAPDH) in the input samples was plotted as mean± SEM. n=4

Table S1. List of PCR primers: Sequences of the PCR primers for genomic PCR amplification, RT-PCR, miRNA, RIP, IVP, and ChIP are listed below. All primers are for human. Related to STAR METHODS.

**Primer Name**	**Primer Sequence**	**Annotation**
DROSHA-in3-F (primer #1)	5'-GCATTTGGAGATGGGAGTG-3'	Genotyping of Drosha locus
DROSHA-in4-R (primer #2)	5'-GGGCAACATAGCGAGATTC-3'	
DROSHA-ex5-R (primer #3)	5'-GGAAGGGTACAAAGTCTGGTCG-3'	
gRNA1	5'-GGAACCCAGTATTAAATGGGTGG-3'	guideRNAs for Crispr-Cas9 Drosha gene editing
gRNA2	5'-CCAAACCAAAAGGATCCAGTAGG-3'	
DROSHA-ex3-qPCR-F	5'-ACATATCCAGGCGGAACATC-3'	qRT-PCR for Drosha ΔN-Drosha
DROSHA-ex5-qPCR-R	5'-ATGGTGATCTTCGGTTGTCTC-3'	
DROSHA-qPCR-F	5'- GAAACTTCGCCACCTCCTAGCA-3'	qRT-PCR for Drosha WT
DROSHA-qPCR-R	5'- CTCCACCGTTACTTCTCGTCTC-3'	
DGCR8-qPCR-F	5'- GGTCCGCCCTGTCTATAATTTC-3'	qRT-PCR for Dgcr8
DGCR8-qPCR-R	5'- GAGTCCTCGATGCTGATGTG-3'	
Rps19-qPCR-F	5'- ACTTCAGCCGAGGCTCCAAGAG-3'	qRT-PCR for Rps19
Rps19-qPCR-R	5'- CTCTTTGTCCCTGAGGTGTCAG-3'	
Rps24-qPCR-F	5'- CCAATGTTGGTGCTGGCAAAAAG-3'	qRT-PCR for Rps24
Rps24-qPCR-R	5'- GCACTTCTACCTGCCACACAAC-3'	
Rps26-qPCR-F	5'- GGACATTTCTGAAGCGAGCGTC-3'	qRT-PCR for Rps26
Rps26-qPCR-R	5'- CGATTCCTGACTACTTTGCTGTG-3'	

Rps2-chip-F	5'- GCCACACTGACTAGTTCCTTC-3'	ChIP for Rps2 locus
Rps2-chip-R	5'- CCACTGCCGAAACCTCC -3'	
Rps10-chip-F	5'- GTTCCATCGGCTCCCATC-3'	ChIP for Rps10 locus
Rps10-chip-R	5'- CCCCTACCCCATAAAATAAGCC-3'	
Rpl28-chip-F	5'- TTTTCCCCTCACTCTCATTCG-3'	ChIP for Rpl28 locus
Rpl28-chip-R	5'- ACTGGGAACTTGGGTGAATG-3'	
pre-miR-21-F	5'- TGTCTGCTTGTTTTGCCT-3'	qRT-PCR and RIP assay for miR-21 locus
pre-miR-21-R	5'- GGATATGGATGGTCAGATGAA-3'	
pre-miR-199a-F	5'- GCCAACCCAGTGTTCAGACTA-3'	qRT-PCR for miR-199a
pre-miR-199a-R	5'- GCCTAACCAATGTGCAGACTA-3'	
pri-miR-183/96/182-F	5'- TGAAGGGGAACATTGGCCTC-3'	qRT-PCR and RIP assay for miR-183 cluster locus
pri-miR-183/96/182-R	5'- GGTCATCTCCGAACAGCTCC-3'	
pri-miR-182-F	5'-GCTGGGGAGGCCTCGGTCTGT-3'	qRT-PCR and RIP assay for miR-182 locus
pri-miR-182-R	5'-TGCAGGAAGGACCTTGTCGCA-3'	
pri-let-7b#1-F	5'- TAATACGACTCACTATAGGGACTTCCCAAGACCAGCC-3'	IVP assay for let-7b
pri-let-7b#1-R	5'-GGGGCCAGTTCCAAGTTCATGG-3'	

pri-miR-183-F	5'-TAATACGACTCACTATAGGGATGTGGCCTTCAGGTGGA-3'	IVP assay for miR-183
pri-miR-183-R	5'-TAGGAGCACTCAAGGCAGAAG-3'	
pri-let-b#2-F	5'- CCCTACCTCAGTGACACGAC-3'	RIP assay for let-7b locus
pri-let7b#2-R	5'- ATCTAGCTCCCAGATGCCCA-3'	
pri-miR-3136-F	5'-GTTATGAGCTAGACAAAGGGC-3'	qRT-PCR and RIP assay for miR-3136 locus
pri-miR-3136-R	5'-CTCCTGGACTCAAATGACTCT-3'	
gRNA1	5'-GACCGTAGCAGCCGCTGCTG AGG-3'	guideRNAs for biallelic deletion deleted in the miR-183 hairpin using Crispr-Cas9
gRNA2	5'-AAGTGGGTAAGGTGCTCCGG AGG-3'	
Primer#1 (F1)	5'-CTGCTTGCCTCTCCGAGCCA-3'	Genotyping of pri-miR-183 locus
Primer#1 (F2)	5'-GTCAGTGAATTACCGAAGGGCC-3'	
Primer#1 (R1)	5'-CCAGGCAGTGTAAGGCGATCTG-3'	
Ago2-F	5'-GGGCCCAAGCTTGCCATGTACTCGGGAGCCGGCCCCGCA CTT-3'	To subclone hAgo2(D594) CDS
Ago2-R	5'-CCGCCGGAATTCTCAAGCAAAGTACATGGTGCGCAGAGT-3'	To subclone hAgo2(D594) CDS

Table S2. List of reagents, related to STAR METHODS**.**

#	Reagent	Company	Catalog no.	
01.	Puromycin	InvivoGen	ant-pr-1	
02.	DMEM-high glucose	HyClone lab	SH30022.01	
03.	Lipofectamine2000	Invitrogen	11668-030	
04.	FCS	Hyclone	SH3007103	
05.	Lipofectamine RNAiMax	Invitrogen	13778-150	
06.	SuperSignal™ West Dura extended duration substrate	ThermoFisher	34076	
07.	Nitrocellulose blotting membrane	Genesee Scientific	84-875	
08.	Polybrene	Sigma-Aldrich	TR-1003	
09.	Trypsin	Life technologies	25200-072	
10.	Proteinase K	Invitrogen	P/N100005393	
11.	Protease Inhibitor	Sigma	P8340	
12.	Phosphatase Inhibitor	Sigma	P5726	
13.	Riboprobe System-T7 Kit	Promega	P1440	
14.	ATTO 680	Jena bioscience	NU-821-680	
15.	RNase Inhibitor	Promega	N2111	IVP
16.	RNase inhibitor	Invitrogen	AM2696	RIP
17.	RNeasy Mini kit	Qiagen	74104	
18.	cDNA synthesis kit	Bio-Rad	17088890	
19.	iQ SYBR Green Supermix	Bio-Rad	1708882	
20.	DNase I	Ambion	AM2238	
21.	SDS-PAGE sample buffer	Invitrogen	NP0007	
22.	SDS-PAGE reducing agent	Invitrogen	NP0009	
23.	Dynabeads Protein A	Invitrogen	10002D	
24.	Dynabeads Protein A	Invitrogen	10004D	
25.	MTT	Millipore	CT02	

Table S3. List of antibodies: Following antibodies were used for immunoprecipitation, immunoblot, ChIP, or RIP assay, related to STAR METHODS.

#	Antibody	Company	Catalog no.
01.	Ago2	Cell signaling Technology	2897
02.	Beta-actin	Sigma-Aldrich	A5441
03.	Ddx5	Abcam	ab21696
04.	Dgcr8	Proteintech	10996-1-AP
05.	Drosha	Bethyl	A301-866A
06.	GAPDH	Millipore	MAB374
07.	Gata1	R&D systems	MAB17791-SP
08.	Lamin A/C	Cell signaling Technology	2032
09.	Puromycin	Kerafast	EQ0001
10.	Rpl11	Proteintech	16277-1-AP
11.	Rps19	Santa Cruz Biotechnology	sc-100836
12.	Rps24	Abcam	ab102986
13.	Rps26	Abcam	ab104050
14.	Rpsa	Abcam	ab137388
15.	Smad1	Invitrogen (Thermo Fisher Scientific)	38-5400
16.	p-Smad1/5/8	Cell signaling Technology	9511
17.	IRDye-680RD goat anti-rabbit IgG (H+L)	Li-Cor	926-68071
18.	IRDye-800CW goat anti-rabbit IgG (H+L)	Li-Cor	926-32211
19.	IRDye-680RD goat anti-mouse IgG (H+L)	Li-Cor	926-68070
20.	IRDye-800CW goat anti-mouse IgG (H+L)	Li-Cor	926-32210
21.	anti-Rabbit-IgG-HRP-linked	Cell signaling Technology	7074
22.	anti-Mouse-IgG-HRP-linked	Cell signaling Technology	7076
23.	anti-Rabbit-IgG-HRP-linked	Cell signaling Technology	7077

Table S4. List of miRNA quantitation reagents and siRNAs, related to STAR METHODS.

#	miRNA	Company	Catalog no.
01.	hsa-miR-183 Taqman assay	Applied Biosystems	4427975-002269
02.	has-miR-182 Taqman assay	Applied Biosystems	4427975-002334
03.	hsa-miR-96 Taqman assay	Applied Biosystems	4427975-000186
04.	hsa-miR-21 Taqman assay	Applied Biosystems	4427975-000397
05.	hsa-miR-103 Taqman assay	Applied Biosystems	4427975-000439
06.	hsa-miR-105 Taqman assay	Applied Biosystems	4427975-002167
07.	hsa-miR-199a Taqman assay	Applied Biosystems	4427975-000498
08.	hsa-miR-24 Taqman assay	Applied Biosystems	4427975-000402
09.	hsa-miR-34a Taqman assay	Applied Biosystems	4427975-000426
10.	hsa-miR-330 Taqman assay	Applied Biosystems	4427975-002230
11.	siAgo2	Sigma-Aldrich; ThermoFisher	SASI-Hs02_00343736; n281589
12.	siDgcr8	Sigma-Aldrich	SASI-Hs02_00355944
13.	siDrosha	Dharmacon™	L-016996-00-0005
14.	siControl	Dharmacon™	D-001206-13-05

Table S5. List of Cell lines and plasmids, related to STAR METHODS.

#	Cell line/Plasmid	Company	Catalog no.
01.	HEK 293T cells	ATCC	CRL-3216
02.	ΔN Drosha cells	Generated in-house	N/A
03.	miR-183KO cells	Generated in-house	N/A
04.	lentiCRISPERv2	Addgene plasmid	52961
05.	PMD2.G	Addgene plasmid	12259
06.	psPAX2	Addgene plasmid	12260
07.	pcDNA3.1(+)	Thermo Fisher	V79020

Table S6. Instruments and software, related to STAR METHODS.

no.	Instrument/ software	Experiment	Company	Model no./ version no.
01.	LI-COR	Immunoblot	Odyssey	Odyssey Dlx Imaging System
02.	Bioruptor	Sonication	Diagenode	Bioruptor Pico
03.	Dismembrator/Sonicator	Sonication	Fisher Scientific	550 sonic dismembrator
04.	RT-PCR machine	qRT-PCR	BioRad	CFX connect
05.	NanoDrop spectrometer	Protein, DNA, and RNA quantitation	Thermo Scientific	NanoDrop 2000c
06.	GraphPad Prism	Statistical analysis	GraphPad	Prism 10
